# Achieving ultrahigh electrochemical performance by surface design and nanoconfined water manipulation

**DOI:** 10.1093/nsr/nwac079

**Published:** 2022-04-27

**Authors:** Haisheng Li, Kui Xu, Pohua Chen, Youyou Yuan, Yi Qiu, Ligang Wang, Liu Zhu, Xiaoge Wang, Guohong Cai, Liming Zheng, Chun Dai, Deng Zhou, Nian Zhang, Jixin Zhu, Jinglin Xie, Fuhui Liao, Hailin Peng, Yong Peng, Jing Ju, Zifeng Lin, Junliang Sun

**Affiliations:** College of Chemistry and Molecular Engineering, Beijing National Laboratory for Molecular Sciences, Peking University, Beijing 100871, China; Key Laboratory of Flexible Electronics, and Institute of Advanced Materials, Jiangsu National Synergetic Innovation Center for Advanced Materials, Nanjing Tech University, Nanjing 211816, China; College of Chemistry and Molecular Engineering, Beijing National Laboratory for Molecular Sciences, Peking University, Beijing 100871, China; College of Chemistry and Molecular Engineering, Beijing National Laboratory for Molecular Sciences, Peking University, Beijing 100871, China; Core Labs, King Abdullah University of Science and Technology, Thuwal 23955–6900, Saudi Arabia; College of Chemistry and Molecular Engineering, Beijing National Laboratory for Molecular Sciences, Peking University, Beijing 100871, China; College of Chemistry and Molecular Engineering, Beijing National Laboratory for Molecular Sciences, Peking University, Beijing 100871, China; Electron Microscopy Centre of Lanzhou University, Lanzhou University, Lanzhou 730000, China; College of Chemistry and Molecular Engineering, Beijing National Laboratory for Molecular Sciences, Peking University, Beijing 100871, China; College of Chemistry and Molecular Engineering, Beijing National Laboratory for Molecular Sciences, Peking University, Beijing 100871, China; College of Chemistry and Molecular Engineering, Beijing National Laboratory for Molecular Sciences, Peking University, Beijing 100871, China; College of Chemistry and Molecular Engineering, Beijing National Laboratory for Molecular Sciences, Peking University, Beijing 100871, China; School of Chemical and Environmental Engineering, China University of Mining and Technology, Beijing 100083, China; State Key Laboratory of Functional Materials for Informatics, Shanghai Institute of Microsystem and Information Technology, Chinese Academy of Sciences, Shanghai 200050, China; State Key Laboratory of Functional Materials for Informatics, Shanghai Institute of Microsystem and Information Technology, Chinese Academy of Sciences, Shanghai 200050, China; Key Laboratory of Flexible Electronics, and Institute of Advanced Materials, Jiangsu National Synergetic Innovation Center for Advanced Materials, Nanjing Tech University, Nanjing 211816, China; College of Chemistry and Molecular Engineering, Beijing National Laboratory for Molecular Sciences, Peking University, Beijing 100871, China; Analytical Instrumentation Center, Peking University, Beijing 100871, China; College of Chemistry and Molecular Engineering, Beijing National Laboratory for Molecular Sciences, Peking University, Beijing 100871, China; College of Chemistry and Molecular Engineering, Beijing National Laboratory for Molecular Sciences, Peking University, Beijing 100871, China; School of Physical Science and Technology, Electron Microscopy Centre of Lanzhou University, and Key Laboratory of Magnetism and Magnetic Materials of the Ministry of Education, Lanzhou University, Lanzhou 730000, China; College of Chemistry and Molecular Engineering, Beijing National Laboratory for Molecular Sciences, Peking University, Beijing 100871, China; College of Materials Science and Engineering, Sichuan University, Chengdu 610065, China; College of Chemistry and Molecular Engineering, Beijing National Laboratory for Molecular Sciences, Peking University, Beijing 100871, China

**Keywords:** two-dimensional material, MXene, nanoconfined water, energy storage mechanism, supercapacitors

## Abstract

The effects of nanoconfined water and the charge storage mechanism are crucial to achieving the ultrahigh electrochemical performance of two-dimensional transition metal carbides (MXenes). We propose a facile method to manipulate nanoconfined water through surface chemistry modification. By introducing oxygen and nitrogen surface groups, more active sites were created for Ti_3_C_2_ MXene, and the interlayer spacing was significantly increased by accommodating three-layer nanoconfined water. Exceptionally high capacitance of 550 F g^–1^ (2000 F cm^–3^) was obtained with outstanding high-rate performance. The atomic scale elucidation of the layer-dependent properties of nanoconfined water and pseudocapacitive charge storage was deeply probed through a combination of ‘computational and experimental microscopy’. We believe that an understanding of, and a manipulation strategy for, nanoconfined water will shed light on ways to improve the electrochemical performance of MXene and other two-dimensional materials.

## Teaser

Record-breaking electrochemical energy storage performance achieved in titanium carbides (MXenes) by surface design and nanoconfined water manipulation, with its atomic mechanism revealed.

## INTRODUCTION

Supercapacitors (SCs) are expected to possess both high energy density and power density, together with the metrics of prolonged cycle life and low cost [1,2]. Electrical double-layer (EDL) capacitors, such as commercial carbon electrode materials, have excellent rate performance but unsatisfactory mass-specific/volumetric capacitance [3,4]. In contrast, most pseudocapacitive materials exhibit high specific capacitance, but are limited by their high cost or poor durability [5–8]. Two-dimensional transition metal carbides (MXenes) have been considered as fascinating materials for pseudocapacitors, having both high volumetric capacitance and power density thanks to their highly redox surface, conductive carbide core and rapid ionic transport through interlayer water [9,10].

Titanium carbide (Ti_3_C_2_T*_x_*), the first reported and the best-studied MXene, has also exhibited state-of-the-art electrochemical performance. Through electrode morphology design, high volumetric capacitance (∼1300 F cm^–3^ at 100 mV s^–1^) has been achieved in Ti_3_C_2_T*_x_* hydrogels [10] while high-rate performance (∼200 F g^–1^ at 10 V s^–1^) has been obtained in macroporous or vertically aligned Ti_3_C_2_T*_x_* MXene films [10,11]. Through composites with carbons [12], polymers [13] and transition metal oxides/hydroxides [14,15], inspiring progress was also made with regard to better energy storage applications. This exciting work urgently requires an understanding of charge storage and water confinement in MXenes, and thus the pathways for further enhancing performance.

Recent work has revealed that the capacitance of Ti_3_C_2_T*_x_* MXene in acidic electrolytes mainly originates from the surface redox reaction with non-negligible EDL-like capacitance [16,17]. Additional work further predicted that increasing the proportion of terminal Ti-O or introducing heteroatoms could enhance the capacitance of Ti_3_C_2_T*_x_* MXene [18,19]. On the other hand, experimental and computational approaches studied water confinement and revealed its role in capacitive performance in neutral electrolytes [20,21]. In acidic electrolytes, where superior capacitance was achieved, proton redox and transport in MXene-confined water were also theoretically investigated [22]. However, the literature did not often give a complete picture of how the interactions of surface chemistry, protons and nanoconfined water contribute to the high capacitance and high-rate performance. In addition, many researchers ignored the effect of nanoconfined water when modifying MXenes, probably due to the difficulties in characterization [23]. As a result, manipulating nanoconfined water in between MXene layers to maximize the electrochemical performance of MXene electrodes is still a formidable task.

Nanoconfined water tends to reside in quasi-integer layers in between MXenes and shows layer-dependent properties as theoretically reported [22]. Increasing the layers of nanoconfined water, for example from two to three, is expected to significantly promote the water mobility and proton diffusion coefficients, and hence the electrochemical performance [22]. Though surface modification by adjusting the proportion of terminal -F, -OH and -O of Ti_3_C_2_T*_x_* MXene has affected the way nanoconfined water resides, most cases did not yield more than two layers of nanoconfined water, and the capacitance enhancement was also unsatisfactory [17,18]. Nitrogen doping has been reported to improve the hydrophilicity of carbon materials [24], hence it is promising in manipulating nanoconfined water in between MXenes. However, current work on nitrogen-doped MXene mainly focuses on electronic structure modulation [19,25,26], omitting an in-depth and systematic study of nanoconfined water.

Herein, a facile yet effective method is proposed to design surface-modified Ti_3_C_2_T*_x_* MXene with outstanding electrochemical performance. By introducing oxygen and nitrogen surface groups, nanoconfined water can be manipulated for faster charge storage, and also to create more active sites for ultrahigh capacitance. *Ex-situ* cryo high-resolution transmission electron microscopy (cryo-HRTEM) and other advanced characterization methods combined with theoretical studies unveil the charge storage mechanism and the role of nanoconfined water. With the manipulated three-layer nanoconfined water, the deep active sites are fully exploited and the high-rate performance is retained, contributing to an exceptional volumetric capacitance in a wide range of scan rates.

## RESULTS AND DISCUSSION

### Synthesis and structure analysis

**Figure 1.**
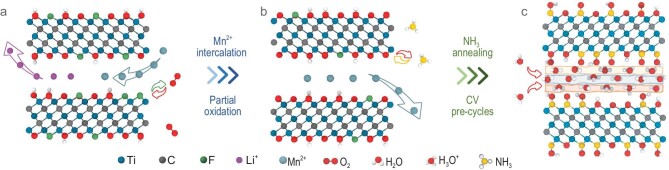
Schematic of modified MXenes synthesis. Structure models of (a) pristine Ti_3_C_2_T*_x_*, (b) Mn^2+^ intercalated and partially oxidized Ti_3_C_2_T*_x_* and (c) further nitrogen-doped Ti_3_C_2_T*_x_*.

The surface-modified Ti_3_C_2_T*_x_* MXene film was prepared by a simple and controllable two-step process: first electrostatic assembly with redox metal cations followed by ammonia treatment (Fig. 1 and Supplementary Fig. S1).

**Figure 2.**
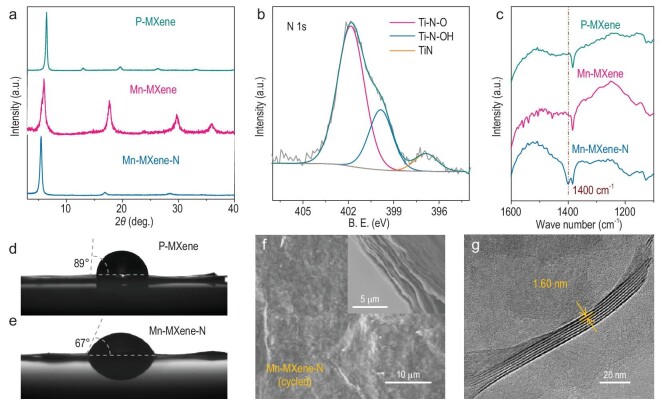
Morphological and structural analysis of Mn-MXene-N. (a) *Ex-situ* XRD patterns of P-MXene, Mn-MXene and Mn-MXene-N films with the interlayer spacing of 14.0 Å, 14.7 Å and 16.2 Å, respectively. (b) XPS results of N 1s of Mn-MXene-N film. (c) The FTIR spectra of the indicated films. The contact angles of (d) P-MXene and (e) Mn-MXene-N. (f) SEM images of the surface and cross-section morphologies of Mn-MXene-N after cycling. (g) Cryo-HRTEM image of Mn-MXene-N.

To lower the energy barrier for nitrogen doping [19] and facilitate the entrance of ammonia, we firstly used cation intercalation to modify the surface chemistry and enlarge the interlayer spacing of Ti_3_C_2_T*_x_* MXene. Outstanding among a series of intercalated cations, Mn^2+^ was selected as a mild cation to intercalate Ti_3_C_2_T*_x_*, and a flexible film (Mn-MXene) was obtained through vacuum filtration. By contrast, other cation-intercalated films (including Fe^2+^, Co^2+^, Ni^2+^, Cu^2+^ and K^+^) were fragile and less conductive, probably due to the uncontrollable structure damage (Supplementary Figs S2–S4, Table S1). Mn-MXene shows a broader (002) peak at a lower angle compared to the pristine Ti_3_C_2_T*_x_* film (P-MXene) in the *ex-situ* X-ray diffraction (XRD) pattern, indicating Mn cation intercalation has enlarged the interlayer spacing (from 14.0 Å to 14.7 Å) and led to clay-like swelling (Fig. 2a and Supplementary Fig. S3). Moreover, the surface chemistry of Ti_3_C_2_T*_x_* was also modulated by redox Mn ion with the assistance of atmospheric oxygen, resulting in a higher proportion of terminal -O and less -F, as evidenced by X-ray photoelectron spectroscopy (XPS) and X-ray fluorescence analysis (Supplementary Fig. S5, Tables S2 and S3). Nitrogen was then introduced into Mn-MXene, and P-MXene for comparison, through ammonia annealing. The interlayer spacing of the final film (Mn-MXene-N) was further enlarged (from 14.7 Å to 16.2 Å, *ex situ* XRD, Fig. 2a), with considerable nitrogen species doping. The nitrogen content of Mn-MXene-N was almost twice that in P-MXene-N, indicating the necessity of cation intercalation pretreatment (elemental analysis, Supplementary Table S4).

The chemical formula of Mn-MXene-N was determined to be Mn_0.12_-Ti_3_C_1.97_F_0.25_O_1.0_(OH)_0.4_-N_0.33_, according to the XPS, X-ray fluorescence spectrum, inductive couple plasma (ICP) and thermogravimetric analysis (Supplementary Tables S2–S5, Figs S6 and S7).

The chemical states of nitrogen were analyzed to be Ti-N-O, Ti-N-OH and Ti-N (Supplementary Discussion 1), with the corresponding N 1s peak position in the XPS (Fig. 2b) located at 401.6, 399.7 and 396.7 eV, respectively. Fourier transform infrared reflection (FTIR) spectra (Fig. 2c, Supplementary Fig. S8) further confirm the existence of Ti-N-O (1400 cm^–1^). This type of nitrogen-doped surface is proven to be more hydrophilic theoretically and experimentally, and can make aqueous electrolyte and the Mn-MXene-N surface contact closer, which is beneficial for double electrode layer energy storage (Supplementary Fig. S9a–c, Fig. 2d and e). The scanning electron microscopy (SEM) images (Supplementary Fig. S10a, Fig. 2f) of Mn-MXene-N before and after the durability test show that the surface and cross section morphologies are smooth and compact, respectively. Furthermore, *ex-situ* cryo-HRTEM and rotation electron diffraction (RED) were performed to obtain additional structural information while preserving the interlayer water. The real space image of Mn-MXene-N (Fig. 2g) shows a uniform interlayer spacing of 16.0 Å (cell parameter *c* = 32.0 Å), consistent with 16.2 Å from the *ex-situ* XRD. The reconstructed 3D reciprocal lattice (Supplementary Fig. S10b–d) of Mn-MXene-N from the RED data gives the cell parameter *a* or *b* with 2.8 Å, close to that of the MAX phase Ti_3_AlC_2_ [27].

### Electrochemical energy storage performance

**Figure 3.**
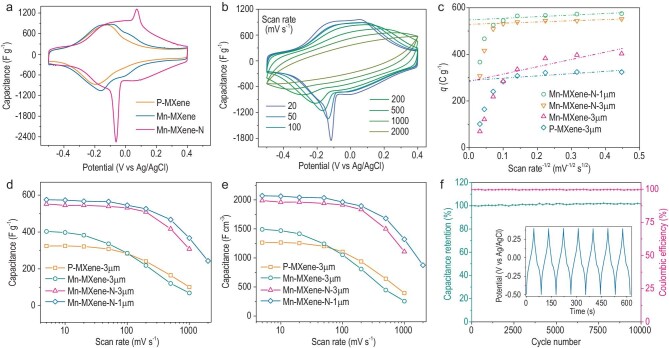
Electrochemical analysis and performance of Mn-MXene-N electrodes. (a) Cyclic voltammograms of the indicated samples at a scan rate of 5 mV s^−1^ in the potential range of −0.5 V to 0.4 V versus Ag/AgCl. (b) Cyclic voltammograms for a 1-μm-thick Mn-MXene-N film at scan rates from 20 mV s^−1^ to 2000 mV s^−1^. (c) Stored charge of 1-μm-thick and 3-μm-thick Mn-MXene-N, 3-μm-thick P-MXene and Mn-MXene electrodes versus root-inverse scan rate, *v*^−1/2^, with *v* from 5 to 1000 mV s^−1^. (d) Gravimetric and (e) volumetric rate performance of the modified films as well as pristine MXene films. (f) Capacitance retention test of Mn-MXene-N electrode. Inset shows the galvanostatic cycling data collected at 10 A g^−1^.

Electrochemical performances of the as-prepared film electrodes are characterized by a three-electrode configuration in 3M H_2_SO_4_. Cyclic voltammograms (CV) of Ti_3_C_2_T*_x_* MXene films with different modifications were tested at 5 mV s^–1^ (Fig. 3a). Among them all, the 3-μm-thick Mn-MXene-N film has the largest integral area in the CV profiles, giving an ultrahigh specific capacitance up to 550 F g^–1^ with corresponding volumetric capacitance reaching an unmatched ∼2000 F cm^–3^. Mn cation intercalation (Mn-MXene) or nitrogen doping (P-MXene-N) can also enhance the specific capacitance of Ti_3_C_2_T*_x_* independently, but only leads to a similar value of ∼400 F g^–1^ (Fig. 3a, Supplementary Fig. S11a). These results indicated the synergistic effects of Mn^2+^ intercalation and nitrogen doping in terms of improving the performance of Mn-MXene-N. The galvanostatic charge/discharge curves in Supplementary Fig. S11b suggest that Mn-MXene-N has a much longer charge/discharge time at 1 A g^–1^ with a quasi-triangular shape, confirming its excellent specific capacitance.

The CV profiles (Fig. 3b) of 1-μm-thick Mn-MXene-N film were then collected at scan rates from 20 mV s^–1^ to 2000 mV s^–1^. The thinner film was used here to minimize ion transport limitations. The shapes of the CVs were well maintained below 500 mV s^–1^ and a considerable integral area still remained even at 2000 mV s^–1^. To analyze the charge storage kinetics of Mn-MXene-N, the Trassatti analysis method [28] was used here, in which the total amount of stored charge (*q*_T_) contained non-diffusion-controlled charges (*q*_n_, independent of scan rates (*v*)) and diffusion-controlled charges (*q*_d_, proportional to *v*^–1/2^). The non-diffusion-controlled and total charges can be obtained by analyzing the dependence of the measured specific charge (*q*) on *v*^−1/2^ and 1/*q* on *v*^1/2^ (Fig. 3c, Supplementary Fig. S12). The *q*_n_ was found to dominate in 3-μm-thick and 1-μm-thick Mn-MXene-N, accounting for ∼94% of *q*_T_, exceeding 470 C g^–1^ while *q*_n_ accounted for 82% and 56% in the *q*_T_ of 3-μm-thick P-MXene and Mn-MXene, respectively, not exceeding 250 C g^–1^. These results show the potential high-rate performance of Mn-MXene-N, which is further demonstrated by the following gravimetric and volumetric rate tests.

As shown in Fig. 3d and e, 3-μm-thick Mn-MXene-N delivered an exceptional capacitance exceeding 500 F g^–1^ (∼1800 F cm^–3^) at scan rates from 5 mV s^–1^ to 200 mV s^–1^, with a decay <10%. Even at a high scan rate up to 1000 mV s^–1^, a good capacitance of 300 F g^–1^ (∼1100 F cm^–3^) was still obtained. To the best of our knowledge, these results surpass work based on all kinds of materials in volumetric capacitance (Supplementary Discussion 2, Fig. S13a). Mn-MXene-N showed a thickness-dependent electrochemical performance due to the limitation of vacuum filtration. Nonetheless, admirable volumetric capacitance was still obtained with a thickness of 16 μm (5 mg cm^–2^) and 36 μm (10 mg cm^–2^) (Supplementary Fig. S13b–d). It is believed that the intrinsic properties of our MXenes could be fully utilized when combined with state-of-the-art electrode morphology. Moreover, the capacitance of Mn-MXene-N shows ∼100% retention and coulombic efficiency when repeating the charge/discharge tests at 10 A g^–1^ for 10 000 cycles in air (Fig. 3f).

As far as is known, this combination of ultrahigh volumetric capacitance and outstanding rate performance in Mn-MXene-N is very rare in electrochemical energy storage (EES) materials. The reason can be attributed to the dilemma presented by the fact that improving ion transport of EES materials has been mainly achieved through increasing surface area, inevitably sacrificing the volumetric capacitance [23]. Nevertheless, another way to significantly accelerate proton transport in a minimum volume was explored by manipulating the nanoconfined water and simultaneously introducing extra active sites by modifying the surface chemistry, which together contribute to the ultrahigh performance of Mn-MXene-N as discussed below.

### The origin of increased capacitance

To identify the origin of the increased capacitance and probe the charge storage mechanism, a preliminary investigation was initially undertaken based on the electrochemical analysis. The CV profiles (Fig. 3a) showed that Mn-MXene has similar redox peaks to P-MXene near -0.15 V versus Ag/AgCl, with a slightly higher current density. In addition, a new pair of redox peaks appeared at 0 V, which was also reported in pristine Ti_3_C_2_T*_x_* under a very low scan rate [16] or in vertically aligned electrodes [11]. These redox peaks were independent of Mn ions, since the cations would be lost due to ion exchanges after pre-cycles (ICP, Supplementary Table S5). The role of Mn^2+^ intercalation was to facilitate the surface modification of Ti_3_C_2_T*_x_*, i.e. increase the proportion of -O (XPS, Table S2), thus enhancing the pseudocapacitance as predicted in previous work [16,18]. In the case of Mn-MXene-N (Fig. 3a), another pair of redox peaks appeared in the potential range from −0.1 V to 0.1 V, which had a larger peak separation when compared to that in P-MXene or Mn-MXene, implying that the introduction of N species might create new active sites. The current density of Mn-MXene-N film above 0.1 V was also obviously increased, where the electrochemical behavior was supposed to be capacitive (Supplementary Fig. S15).

**Figure 4.**
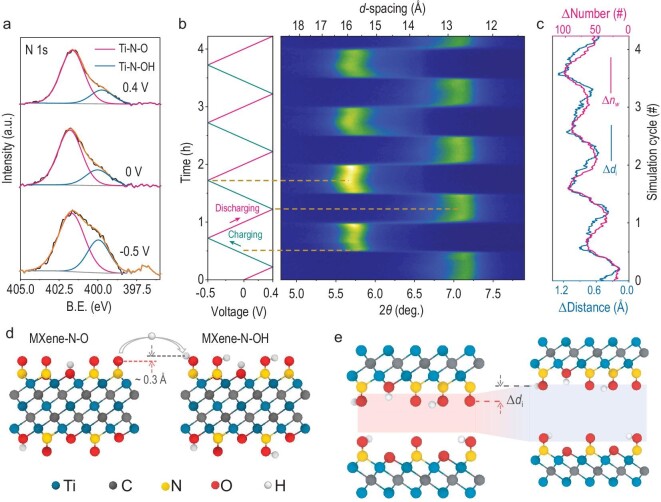
Surface evolution and interlayer spacing change of Mn-MXene-N during CV test. (a) *Ex-situ* XPS spectra of N 1s for Mn-MXene-N after holding at different potentials. The proportion of Ti-N-O is reduced with the applied potential decreased. (b) *In-situ* XRD patterns during CV cycles at 0.5 mV s^–1^. (d) Change of *c* lattice parameter calculated from static DFT. (c and e) Change of the interlayer H_3_O^+^ and H_2_O numbers Δ*n*_w_ and change of the inner surface distance Δ*d*_i_ observed during charge-discharge MD simulations.

To further investigate the capacitance origin of Mn-MXene-N, *in-/ex-situ* experiments combined with theoretical computations were conducted. As shown in the *ex-situ* XPS spectra of N 1s (Fig. 4a, Supplementary Fig. S16), significant enhancement of the peak intensity of Ti-N-OH with a decrease in Ti-N-O was observed when the applied potential was reduced from 0.4 V to −0.5 V, confirming the valence reduction of nitrogen. *Ex-situ* soft X-ray absorption spectroscopy was used to study the valence change of the element Ti. The peak intensity ratio of Ti L_3_ t_2g_ to e_g_ progressively decreased, with the pre-edge region shifting to a higher energy (Supplementary Fig. S17) when the applied potential was increased, suggesting an increased Ti oxidation state [29]. These results also imply that terminal N species could provide extra capacitance without blocking the redox reaction of Ti-O.

Furthermore, an *in-situ* XRD experiment was conducted to trace the interlayer spacing change of Mn-MXene-N during the CV test at 0.5 mV s^–1^ (Fig. 4b, Supplementary Figs S18 and S19). During charging (cathodic polarization), a sharp *d*-spacing transition of ∼2.8 Å at -0.05 V and a gradual increase of ∼0.5 Å in the overall potential range from 0.4 V to -0.5 V were observed. Upon discharging, this process was reversed, indicating that the cycling process was highly reversible. Molecular dynamic (MD) simulation and density functional theory (DFT) calculations were conducted to investigate the interlayer spacing change induced during the charging/discharging process and by the transmission from Ti-N-O to Ti-N-OH. The MD simulations observe a reversible intercalation/deintercalation of H_3_O^+^ and H_2_O between the MXene layers during the charging/discharging process, which directly leads to an increase/decrease in the interlayer spacing. The change of interlayer H_3_O^+^ and H_2_O numbers, Δ*n*_w_, exhibited a high correlation with the change of the inner surface distance Δ*d*_i_ (Fig. 4c and e), with the average normalized correlation coefficient reaching ∼96.8%. Also, the static DFT calculation shows a ∼0.6 Å interlayer spacing change (0.3 Å × 2, on both sides of MXene) when the Ti-N-O transforms to Ti-N-OH (Fig. 4d).

The high correlation between the Δ*n*_w_ and Δ*d*_i_ qualitatively revealed that the sharp change of interlayer spacing up to 2.8 Å originated from sudden H_3_O^+^/H_2_O intercalation at the corresponding potential. When considering that the intercalated number of H_2_O is a tenth that of H_3_O^+^, it is reasonable to mainly attribute water intercalation to the interlayer spacing change (Supplementary Figs S20 and S21). This larger interlayer spacing change of Mn-MXene-N allows the in-layer water structure to directly transform from one layer to three layers, indicating that more H_3_O^+^/H_2_O could be involved in the charging and discharging process, leading to a higher net charge (n_q_) storage. These results highlight how nanoconfined water manipulation can maximize electrochemical performance, which will be discussed below with experimental and computational evidence.

Moreover, it was noticed that the specific capacitance of Mn-MXene-N above 0.05 V was considerably higher than P-MXene (Fig. 3a). In this potential range, the charge storage behavior was inferred to be EDL-like, in which the capacitance is determined by the formula $C\ = {\varepsilon _0}\ {\varepsilon _r}\times A/d$. Though the specific surface areas of all the MXene films were measured to be small (N_2_ adsorption-desorption measurements, Supplementary Fig. S22), it was speculated that the electrochemical surface area (A) might actually be large and similar for the three samples, when considering protons are much smaller than N_2_. Under this hypothesis, a stronger de-solvation and a narrower charge separation distance $d_{{\rm{s}} - {\rm{Mn}} - {\rm{MXene}} - {\rm{N}}}^{\rm{q}}$ (∼1.1 Å, Supplementary Fig. S23) spacing would lead to a higher capacitance, when compared with ${\rm{\ }}d_{{\rm{s}} - {\rm{P}} - {\rm{MXene}}}^{\rm{q}}$ (∼2.5 Å), accounting for the higher specific capacitance of Mn-MXene-N above 0.05 V.

### The investigation and manipulation of nanoconfined water

Nanoconfined water in between MXene layers provides a major way for protons to move to and from the redox surface, and hence the properties of nanoconfined water will strongly affect proton transport. Experiments were designed and theoretical calculations conducted to manipulate the nanoconfined water and probe its role in electrochemical performance.

**Figure 5.**
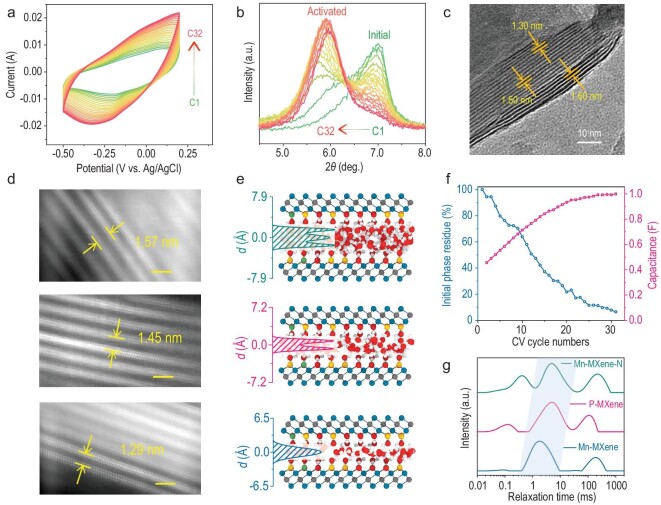
Layer-dependent properties of nanoconfined water. (a) CV curves of Mn-MXene-N during CV pre-cycles at 20 mV s^–1^. (b) *In-situ* XRD patterns and (c) the *ex-situ* cryo-HRTEM image of Mn-MXene-N captured during the CV pre-cycles. (d) The atomic resolution HAADF images of three distinct phases with interlayer spacing of 1.29, 1.45 and 1.57 nm, respectively. All scale bars are 2 nm. The experimental results are highly consistent with MD simulations. (e) Local snapshot of the Mn-MXene-N model with three-layer (15.8 Å), two-layer (14.4 Å) and one-layer (13.0 Å) water in between the layer of MD simulation, with the probability density profiles of water molecules along the *c* lattice direction displayed on the left side. (f) The trends of the initial phase residue and capacitance enhancement of Mn-MXene-N during CV pre-cycles. (g) Low field ^1^H time-domain nuclear magnetic resonance spectra of the indicated samples. The shaded region represents the time-domain region of interlayer water.

Firstly, how the nanoconfined water resides in between Mn-MXene-N was investigated. A common phenomenon was noticed whereby MXenes (and also some 2D and layered materials) usually need dozens of CV pre-cycles, or to rest in the electrolytes, to achieve their best electrochemical performance in aqueous electrolytes, and this is referred to as the activation process. This process is supposed to be a result of water intercalation, though this lacks solid evidence. Thus, *in-situ* XRD was performed during the CV pre-cycles (Fig. 5a) of Mn-MXene-N to investigate this process. As shown in Fig. 5b and Supplementary Fig. S24, the initial phase (partially dehydrated, at 7.0°) was evolved into the activated phase (fully hydrated, at 5.6°) during the activation process, with the corresponding interlayer spacing increased from 12.7 Å to 15.7 Å. The two peaks are distinct, implying that the change of interlayer spacing due to water intercalation might not be continuous.

To provide complementary evidence at the atomic level, *ex-situ* cryo-HRTEM was used to observe the interphase between the initial and activated phase during pre-cycles. As shown in Fig. 5c and Supplementary Fig. S25, a clear interlayer spacing change from 13.0 Å to 16.0 Å was observed, corresponding to the transformation of the initial phase to the activated phase. Surprisingly, in such a small domain, the change of interlayer spacing was still discrete. Moreover, the atomic resolution high-angle annular dark-field (HAADF) images of these distinct phases were also captured individually (Fig. 5d). Atomically imaging water molecules in between MXenes using the current TEM technique was extremely difficult (Supplementary Discussion 3). Thus, we conducted MD simulations to shed light on this unusual phenomenon, which indicated that water molecules tend to be intercalated in quasi-integer layers with particular interlayer spacing (Supplementary Fig. S26). Furthermore, the three phases with interlayer spacing of 12.9 Å, 14.5 Å and 15.7 Å were assigned to be Mn-MXene-N with one, two and three layers of confined water respectively, according to the simulation results (Fig. 5e). The cryo-HRTEM image also indicated a fast transition of the initial phase to the activated phase, which was completed within dozens of nanometers, accounting for the distinct peaks observed in the *in-situ* XRD.

Here, the layer-dependent properties of nanoconfined water (with additional investigations presented in Supplementary Discussion 4) was probed. Firstly, three-layer nanoconfined water in between Mn-MXene-N showed much improved water mobility and proton diffusion coefficient than one-layer water, close to bulk water as evidenced by the MD simulations results (Supplementary Fig. S29). This enhancement of ionic mobility could boost the charging and discharging process and effectively shorten its charging time, which is consistent with the calculated time constant (τ) of resistance capacitance transmission line model equivalent circuit (Supplementary Fig. S21). In addition to the kinetic perspective, the increased layer spacing could markedly reduce the free energy barrier (ΔG) for H_3_O^+^/H_2_O migrating from the bulk electrolyte into the MXene galleries via the edges of the MXene layers, while the ΔG for three layer case is 2.5 kJ mol^–1^, smaller than the ΔG of 3.5 kJ mol^–1^ for one layer case. Meanwhile, the calculated hydrogen bonding number density ρ_H-bond-3layer_ in the three-layer water structure is ∼29.2 #/nm^3^, while ρ_H-bond-1layer_ in one-layer water is only ∼10.2 #/nm^3^. This enhanced denser hydrogen bonding network could effectively promote the proton-hopping transport, likely via the Grotthuss mechanism. The reduction of the energy barrier and the enhanced denser hydrogen bonding network jointly provide a solution to the main bottleneck for the charging process [22].

Thus, superior electrochemical performance in activated MXenes is expected and confirmed by *in-situ* experiments. In the *in-situ* XRD during the activation process, it was noticed that the peak intensity of the initial phase gradually decreased and further disappeared in the end, with the obtained activated phase appearing in the reverse trend. An enhancement of the integral area of CVs was simultaneously observed (Fig. 5a). When combining the two trends, an interesting result was produced: the increased specific capacitance during activation was highly correlated with the proportion of the initial phase converted (Fig. 5f, Supplementary Fig. S30). This semi-quantitative relationship suggested that the specific capacitance of Mn-MXene-N can be fully exploited with three-layer water, thereby nearly doubling the capacitance of the initial phase.

*In-situ* electrochemical impedance spectroscopy (EIS) was also performed to investigate the effect of water intercalation. As illustrated in Supplementary Fig. S31, an obvious decrease of the 45-degree linear part (related to ion transport resistance) and a steeper slope of the Nyquist plot in the low-frequency range (related to specific capacitance) were observed when the CV cycle numbers were increased, indicating the fruitful role of intercalated water on both ion accessibility and the exploitation of active sites.

In addition, in the potential range (below 0.05 V versus Ag/AgCl) that pseudocapacitance dominates, Mn-MXene-N can accommodate three-layer confined water while P-MXene can only accommodate two layers, as shown in this work and a previous work [16]. Hence, protons and water in between Mn-MXene-N layers can be transported more quickly, leading to a superior rate performance (Supplementary Fig. S32). It was also noticed that Mn-MXene has a worse rate retention than P-MXene despite both having two-layer water. This may be attributed to other factors affecting proton mobility, including surface terminals, intercalated cations and defects [30–32]. Nevertheless, layers of nanoconfined water are still the dominant factor, which was experimentally confirmed by low field ^1^H nuclear magnetic resonance.

Figure 5g depicts the continuous distribution of the spin-spin relaxation time, T2, for the three samples. Generally, there are three recognizable states of protons in these films, that is, protons in bonded terminals, interlayer water and free water, based on the feature that protons with higher mobility have a longer relaxation time. The time-domain region between 0.5 ms and 20 ms is ascribed to interlayer water (including hydrated proton). In this region, Mn-MXene film has the lowest relaxation time of all, and is responsible for the worst rate performance. Mn-MXene-N film has the longest relaxation time of all, and further with higher mobility of protons in meso-split water (time-domain near 100 ms) and freer surface proton (time-domain below 0.5 ms), which together account for the much better rate performance of Mn-MXene-N. On the other hand, MD simulations indicate that Mn-MXene-N with three-layer confined water can store more charge and simultaneously accelerate the charging/discharging process (Supplementary Fig. S21), further confirming the superior electrochemical performance. Unlike conventional methods that increase porosity, another way was demonstrated here to maximize the capacitive performance of Ti_3_C_2_T*_x_* MXene through manipulating the behavior of confined water, in which high volumetric capacitance of bulk layered materials was perfectly combined with the fast ion diffusion of exfoliated 2D materials.

## CONCLUSION

This work shows how surface chemistry modification and nanoconfined water manipulation can enhance electrochemical performance (improving its specific capacitance and rate performance, simultaneously). The designed Mn-MXene-N delivers an ultrahigh volumetric capacitance at wide scan rates (above 1800 F cm^–3^ at 200 mV s^–1^ and 1100 F cm^–3^ at 1000 mV s^–1^), far superior to most state-of-the-art electrode materials. The origin of the increased capacitance was also investigated and the role of interlayer water in ultrahigh electrochemical performance was understood. In particular, through the first use of cryo-HRTEM in MXene fields associated with other advanced characterization methods and computational simulations, a clear picture of how nanoconfined water resides and acts in MXene layers was achieved during the electrochemical process. Precisely manipulating and understanding the nanoconfined water of MXenes is of wide interest but remains a cutting-edge challenge. Nevertheless, our work can open up opportunities in this field. Moreover, on account of the facile synthesis strategy and fundamental mechanism study, this work might also provide new insights into other 2D and layered materials with nanoconfined fluids beyond MXenes, and be applied beyond energy storage, to areas such as water desalination and ion-selective membranes.

## Supplementary Material

nwac079_Supplemental_File
