# Supplementary material for: Achieving ultrahigh electrochemical performance by surface design and nanoconfined water manipulation
Source: Natl Sci Rev. 2022 Apr 27;9(6):nwac079. doi: 10.1093/nsr/nwac079 (PMC9166535; doi:10.1093/nsr/nwac079)
Supplement: nwac079_Supplemental_File [file nwac079_supplemental_file.docx]

**Achieving ultrahigh electrochemical performance by manipulating nanoconfined water**

Haisheng Li^1,^ ^†^, Kui Xu^2,^ ^†^, Pohua Chen^1^, Youyou Yuan^1,3^, Yi Qiu^1^, Ligang Wang^1^, Liu Zhu^4^, Xiaoge Wang^1^, Guohong Cai^1^, Liming Zheng^1^, Chun Dai^1,5^, Deng Zhou^6^, Nian Zhang^6^, Jixin Zhu^2^, Jinglin Xie^1,7^, Fuhui Liao^1^, Hailin Peng^1^, Yong Peng^8^, Jing Ju^1^, Zifeng Lin^9^, Junliang Sun^1^*

^1^College of Chemistry and Molecular Engineering, Beijing National Laboratory for Molecular Sciences, Peking University, Beijing 100871, China.

^2^Key Laboratory of Flexible Electronics (KLOFE) & Institute of Advanced Materials (IAM), Jiangsu National Synergetic Innovation Center for Advanced Materials (SICAM), Nanjing Tech University (NanjingTech), Nanjing 211816, China.

^3^Core Labs, King Abdullah University of Science and Technology (KAUST), Thuwal 23955–6900, Saudi Arabia.

^4^Electron Microscopy Centre of Lanzhou University, Lanzhou University, Lanzhou 730000, China

^5^School of Chemical and Environmental Engineering, China University of Mining and Technology, Beijing 100083, China.

^6^State Key Laboratory of Functional Materials for Informatics, Shanghai Institute of Microsystem and Information Technology, Chinese Academy of Sciences, Shanghai 200050, China.

^7^Analytical Instrumentation Center, Peking University, Beijing 100871, China.

^8^School of Physical Science and Technology, Electron Microscopy Centre of Lanzhou University and Key Laboratory of Magnetism and Magnetic Materials of the Ministry of Education, Lanzhou University, Lanzhou 730000, China

^9^College of Materials Science and Engineering, Sichuan University, Chengdu, 610065, China.

^†^These authors contributed equally. *e-mail: [junliang.sun@pku.edu.cn](mailto:junliang.sun@pku.edu.cn).

**TABLE OF CONTENTS**

**Supplementary Methods**............................................................................................4

[**Fig. S1.** Illustration of the synthesis of Mn-MXene-N. 7](#_Toc79409483)

[**Fig. S2.** Optical pictures of cations intercalated Ti_3_C_2_T*_x_*. 7](#_Toc79409484)

[**Fig. S3.** XRD patterns of pristine and different cations intercalated Ti_3_C_2_T*_x_*. 8](#_Toc79409485)

[**Fig. S4.** Ti 2p XPS spectra of cation intercalated MXene. 9](#_Toc79409486)

[**Fig. S5.** XPS survey spectra of P-MXene and Mn-MXene. 10](#_Toc79409487)

[**Fig. S6.** TGA analysis of Mn-MXene-N 13](#_Toc79409488)

[**Fig. S7.** XPS survey spectra of Mn-MXene-N**.** 13](#_Toc79409489)

[**Fig. S8.** The FTIR spectra of P-MXene, Mn-MXene and Mn-MXene-N. 14](#_Toc79409490)

[**Fig. S9.** Snapshot of surface water droplet hydrophilic process. 16](#_Toc79409491)

[**Fig. S10.** SEM images and RED data of MXenes. 17](#_Toc79409492)

[**Fig. S11.** Cyclic voltammograms and Galvanostatic charging/discharging profiles.. 18](#_Toc79409493)

[**Fig. S12.** Inverse of stored charge versus the square root of the scan rate.. 18](#_Toc79409494)

[**Fig. S13.** Comparison of the volumetric capacitance. 19](#_Toc79409495)

[**Fig. S14**. CV profiles of the other work compared to this work 21](#_Toc79409496)

[**Fig. S15.** EIS at different potentials and CV at 2 to 200 mV s-1 of Mn-MXene-N. 22](#_Toc79409497)

[**Fig. S16.** *Ex-situ* XPS spectra of N 1s for Mn-MXene-N after holding at different potentials. 23](#_Toc79409498)

[**Fig. S17.** *Ex-situ* Ti L-edge XAS spectra of Mn-MXene-N. 23](#_Toc79409499)

[**Fig. S18.** The schematic of modified in situ XRD cell and working electrode. 24](#_Toc79409500)

[**Fig. S19.** Cyclic voltammogram of Mn-MXene-N at 0.5 mV s−1 in in-situ XRD 26](#_Toc79409501)

[**Fig. S20.** Schematic of the simulation model of MXene-based supercapacitors... 26](#_Toc79409502)

[**Fig. S21.** The intercalation/deintercalation numbers of H_2_O, H_3_O^+^ and SO_4_^2-^ (HSO_4_^2-^) during charging/discharging process. 27](#_Toc79409503)

[**Fig. S22.** Dynamic simulations of MXenes during charging /discharging. 27](#_Toc79409504)

[**Fig. S23.** N2 adsorption-desorption isotherms and pore-size distributions.**.** 28](#_Toc79409505)

[**Fig. S24.** Calculation of the charge separation distance between the center of charge and the surface of the electrode 29](#_Toc79409506)

[**Fig. S25.** In situ and ex situ XRD patterns of Mn-MXene-N film. 30](#_Toc79409507)

[**Fig. S26.** The average interlayer spacing from cryo-HRTEM images. 30](#_Toc79409508)

[**Fig. S27.** The distribution of confined water with discrete layers under different interlayer spacin 31](#_Toc79409509)

[**Fig. S28.** Comparison of probability profiles of dipole orientation of different layers of confined water inside Mn-MXene-N layers. 33](#_Toc79409510)

[**Fig. S29.** XRD patterns of charged and discharged as well as the cryo-dried Mn-MXene-N 34](#_Toc79409511)

[**Fig. S30.** Water mobility and proton diffusion in MXene confined water layers.. 35](#_Toc79409512)

[**Fig. S31.** Methods for XRD refinement. 36](#_Toc79409513)

[**Fig. S32.** *In-situ* EIS test of Mn-MXene-N during CV pre-cycles*.* 37](#_Toc79409514)

[**Fig. S33.** Capacitance retention of P-MXene, Mn-MXene and Mn-MXene-N. 37](#_Toc79409515)

[**Table S1.** Area resistance (Ω cm-2) of cation intercalated films 9](#_Toc79408097)

[**Table S2.** XPS results of P-MXene and Mn-MXene. 12](#_Toc79408098)

[**Table S3.** XRF analysis of P-MXene and Mn-MXene 12](#_Toc79408099)

[**Table S4.** Elemental analysis of P-MXene-N and Mn-MXene-N 13](#_Toc79408100)

[**Table S5.** ICP results of t Mn-MXene-N film during CV pre-cycles.. 13](#_Toc79408101)

[**Supplementary discussion 1.** The chemical states of doped nitrogen in Ti3C2 MXene 15](#_Toc79408102)

[**Supplementary discussion 2.** The excluded articles when comparing the volumetric capacitance. 21](#_Toc79408103)

[**Supplementary discussion 3.** The difficulties in atomically imaging water. 31](#_Toc79408104)

[**Supplementary discussion 4.** Discussions on the nature of layered nanoconfined water 32](#_Toc79408105)

**Supplementary Methods**

**Chemicals** FeSO_4_·7H_2_O was supplied from Xilong Scientific Co., Ltd. Co(OAC)_2_·4H_2_O, Cu(OAC)_2_·H_2_O, Ni(OAC)_2_·4H_2_O and Mn(OAC)_2_·4H_2_O were purchased from Beijing Chemical Works. KOH was obtained from Sinopharm Chemical Reagent Co., Ltd. LiF and HCl were provided by Beijing Tongguang fine chemicals company. Ti_3_AlC_2_ was purchased from 11 technology Co., Ltd. All the chemicals were used as received without further purification. The Milli-Q water (18.2 MΩ, Millipore) was used in all experiments.

**Characterization.** The morphologies and structure of all samples were examined by SEM (Hitachi S-4800), TEM (JEM-2100) and high-resolution TEM (JEM-2100F, acceleration voltage 200 kV). The atomic-resolution STEM images were performed on a probe aberration-corrected STEM (Themis TitanG2 80-300, FEI, USA) operated at 300 kV. Elemental distributions were analyzed by an energy dispersive spectrometer (EDS, Bruker Xflash 6100) at an acceleration voltage of 15 kV. Powder X-ray diffraction data were recorded using a Rigaku D/Max-2000 diffractometer with graphite monochromatized Cu Kα radiation (λ = 0.15406 nm) in a step of 0.125°/min under the tube conditions 40 kV and 100 mA. Chemical compositions and oxidation state of the samples were further analyzed using high-resolution XPS with monochromated Al Kα radiation (hν = 1486.6 eV), Binding energies were referenced to the C 1s peak of (C-C, C-H) bond, which was set at 284.8 eV. The peak fitting was carried out using commercially available software, CasaXPS. The ratio of Mn to Ti was determined using inductively coupled plasma mass spectrometry (ICP-OES, Agilent 7500C). The measurements of Ti to F ratio were performed on a Zetium XRF instrument from Malvern Panalytical. SuperQ software (including Virtual Analyst) was used for data collection and analysis. Each element was identified by the peak corresponding to its known Kα energy. Fourier transform infrared spectroscopy (FT-IR) spectra were analyzed by a Spectrum Spotlight 200 FT-IR microscope with an attenuated total reflection accessory (Spotlight 200, PerkinElmer, USA). Before conducting FTIR experiments, the samples, as well as KBr, were treated under 120 ^o^C to remove the adsorbed water. TG was studied using a Netzsch Libra TG209 F1 thermogravimetric analyzer. The *ex-situ* Ti L-edge XAS data were collected in total fluorescence yield (TFY) mode, which was carried out under ultrahigh vacuum (10-9 torr) in a single load at room temperature using beamline BL02B02 of SSRF.

**Synthesis of cation intercalated Ti_3_C_2_T_x_.** The few-layer Ti_3_C_2_T*_x_* was synthesized from Ti_3_AlC_2_ through a mixture of hydrochloric acid (HCl) and lithium fluoride (LiF), which was similar to a previous report^1^. The obtained few-layer Ti_3_C_2_T*_x_* supernatant was stored at 4 °C in the refrigerator after purging N_2_ gas for 0.5 h. For the synthesis of cation intercalated Ti_3_C_2_T*_x_*, typically, 0.74 g Mn(OAC)_2_·4H_2_O was dispersed into 20 mL of deionized water and stirred for 10 min. Then, 20 mL Ti_3_C_2_T*_x_* supernatant (~1.0 mg mL^-1^) was added into the solution and further stirred for 4 h with the mixed solution exposed to air. Since MXene surface is negatively charged, positively charged cations can be spontaneously intercalated into MXene layers due to electrostatic attraction^2^. Prolonged intercalated time would improve the surface modification effect but would also lead to structural damage. The mixed solution finally resulted in sediments due to electrostatic assembly. The sediments were vacuum-filtered to obtain free-standing film through nanoporous polypropylene membrane (0.22 um). The obtained film was denoted as Mn-MXene film. By contrast, 0.84 g FeSO_4_·7H_2_O, 0.75 g Co(OAC)_2_·4H_2_O, 0.6 g Cu(OAC)_2_·H_2_O, 0.75 g Ni(OAC)_2_·4H_2_O, and 0.19 g KOH were used to synthesize corresponding cation intercalated films in a similar method. And their reaction time is limited to 4 hours. 20 mL Ti_3_C_2_T*_x_* supernatant (1.0 mg mL^-1^) was directly vacuum-filtered to obtain pristine Ti_3_C_2_T*_x_* film (denoted as P-MXene film).

**Synthesis of nitrogen-doped Ti_3_C_2_T*_x_*.** The nitrogen-doped Ti_3_C_2_T*_x_* was obtained by annealing P-MXene and Mn-MXene films in 200 ml min^−1^ ammonia gas flow at 350 °C for 6 h in a tube furnace, and the resulting samples were labeled as P-MXene-N and Mn-MXene-N respectively.

**Electrochemical measurements.** All electrochemical tests were performed using an electrochemical analyzer, CHI 660E, in a 3 M H2SO4 aqueous electrolyte and under ambient conditions. The flexible films were directly used as working electrodes and glassy carbon was used as charge collector. In the three-electrode configuration, flexible films, over capacitive activated carbon (or carbon rod), and Ag/AgCl in 3 M KCl were used as working, counter, and reference electrodes, respectively. Cyclic voltammograms (CV) tests and galvanostatic charge-discharge (GCD) tests were performed in the range of -0.5 to 0.4 V. Electric impedance spectroscopy (EIS) was performed with an amplitude of 5 mV, from 100 mHz to 100 kHz. EIS spectra collected at various potentials were recorded after holding at each potential for 5 minutes. Cycling stability measurement was performed by repeating the constant current charge/discharge at 10 A g^-1^ for 10000 cycles. The open circuit voltage (OPCV) of Mn-MXene-N was measured to be 0.12 V *vs* Ag/AgCl. We always conducted CV pre-cycles before any performance evaluation.

***Ex-situ* characterizations.** Since interlayer water has a strong effect on the electrochemical performance of MXenes, we conducted *ex situ* characterizations in some cases to prevent the loss of interlayer water and to provide more precise information of the materials. In a typical *ex-situ* characterization, the MXene film was firstly fully activated through CV pre-cycles and then was charged to -0.5 V versus Ag/AgCl. After that, the film was washed by deionized water or ethanol and was used for particular characterizations (XRD, cryo-HRTEM and NMR) as soon as possible (usually within 15 minutes).

***In situ* XRD.** The flexible Mn-MXene-N film is directly connected to the copper wire with the contact point covered with silver glue. The silver and exposed copper wire were coated with epoxy resin adhesive (Fig. S13b). A similar three-electrode configuration with free-standing Mn-MXene-N film served as the working electrode was used for *in situ* XRD test in a modified 3-electrode cell, as represented in Fig. S13a. Cyclic voltammetry in the potential range from -0.5 V to 0.2 V at 20 mV s^−1^ (for the activation experiment) and -0.5 V to 0.4 V at 0.5 mV s^−1^ (for the reversible interlayer spacing change test) were performed to control the working electrode potential, respectively. XRD patterns of the Mn-MXene-N electrode during cycling was collected on a PANalytical powderX diffractometer using a Cu Kα radiation (λ = 1.5406 Å), Bragg-Brentano geometry and a PIXEL1D detector in the range 2θ = 4–8° with a step of 0.01°.

**Low field ^1^H NMR.** This technique by measuring proton relaxation time is more sensitive to the confinement rather than the chemical state, which is used to detect the mobility and content of proton^3^. In NMR experiments, the applied magnetic field was set at 0.5 T with the corresponding resonance frequency for ^1^H at 23.311 MHz in a low-field nuclear magnetic resonance spectrometer (MesoMR, Niumag Corporation, Shanghai, China). The mass of the MXene film is around 20 mg. The spin-spin relaxation time (T2) of MXene films was collected with the pulse width of 3 μs, sequence repetition time of 5 s and the dwell time between data of 5 μs by Carr-Purcell-Meiboom-Gill sequences. Niumag NMR inverse software was used to analyzed the collected NMR data.

**Cryo-TEM.** (a) Observing the interphase existed in activation process: The as-prepared Mn-MXene-N film served as the working electrode was used for the CV test at the scan rate of 50 mV s^-1^ for 10 cycles. Then the film was immediately was washed by ethanol and ground through an agate mortar with the existence of ethanol. Afterward, the resulted solution was transferred to Cu grid without further ultrasonic. The sample preparation procedure should be as fast as possible to prevent self-discharging (no more than 15 minutes). To avoid the loss of interlayer water in vacuum, a TEM cryo-holder (Gatan) was used to load the sample where TEM grids were immersed in liquid nitrogen. A single-tilt liquid nitrogen cooling holder was used to cool the samples to approximately -170 °C to minimize electron beam damage and interlayer water loss. (b) Observing the atomic resolution high-angle annular dark-field (HAADF) image of the three distinct phases: fully charged Mn-MXene-N was used to obtain the interlayer spacing of 15.7 Å while partially activated Mn-MXene-N was used to image the phases with interlayer spacing of 14.5 Å and 12.9 Å. In this experiment, the samples were ground and dispersed in 3M H_2_SO_4_ solution. The mixture was then directly transferred to Cu grid, which was deposited with robust, ultraclean and atomically thin graphene membranes^4^. The *ex-situ* cryo STEM  experiment was carried out by using in-situ cryo-transfer holder (626, GATAN, USA).

**Computation methods.** Fig. S20 shows the molecular simulation system, two types of four-layer Mn-MXene-N and P-MXene electrodes with different initial layer spacing are immersed in the 3 M H_2_SO_4_ electrolytes. The stoichiometric proportion of the functional groups (-F, -O, -OH, -N-O, -N-OH) are estimated according to our experimental results. In order to achieve tracking layer spacing change during the equilibrium and charging/discharging processes, all the layers of the MXene electrodes are kept moveable in the simulation. All of the MD simulations are conducted using the Large-scale Atomic/Molecular Massively Parallel Simulator (LAMMPS) classical molecular dynamics code package^5^. The static DFT calculation of structural parameters are conducted using the DS-PAW software integrated in Device Studio program and the projector augmented wave method with the Perdew-Burke-Ernzerhof (PBE). The schematic atomic models are displayed and rendered with the OVITO software^6^. The bond and non-bond interaction between the atoms are described with the ClayFF force field^7^. The force field parameters, including Lennard-Jones potential, point patrial charges, harmonic bonds and harmonic angles are taken from the previous relevant works^8,9^. The long-range electrostatic interactions are calculated using the particle-particle particle-mesh (pppm) scheme in k-space. The simulations are performed with Canonical ensemble (NVT) under target temperature 300 K, with the integration time step of 1 fs. To investigate the layer spacing changes of electrode layers upon charging/discharging and accompanied structural and dynamic properties of electrolytes, a simulation strategy mimic galvanostatic cycling experiments is employed here. Firstly, all systems are equilibrated with a null net charge on the electrodes for 1.8 ns, which is a long enough time to ensure the structure could adequately converge. And then, the systems undergo three charging/discharging cycles of 0.3 ns each, with net chagre n*_q_* on the electrodes increasing/decreasing linearly with time. The validity of the simulation method and simulation parameters have been proved in the previous works^10-12^.

**Methods for calculation of specific capacities.**

The gravimetric capacitance (𝐶_𝑤𝑡_, F g^-1^) and volumetric capacitance (𝐶_𝑣𝑜𝑙_, F cm^-3^) were calculated by 𝐶_𝑤𝑡_ =∫𝑖𝑑𝑉 /(𝛥𝑉𝑚𝜈) and 𝐶_𝑣𝑜𝑙_ = 𝜌×𝐶_𝑤𝑡_ , respectively, where 𝑉 represents the potential windows, 𝑚 represents the mass of electrode, 𝜈 represents the potential scan rate, and 𝑖 is the current density. The electrode densities 𝜌 for thin films were measured to be about 3.9 g cm^-3^ for P-MXene, 3.7 g cm^-3^ for Mn-MXene and 3.6 g cm^-3^ for Mn-MXene-N. For thick Mn-MXene-N films, the density is decreased to 3.1 g cm^-3^ for 16 μm (5 mg cm^-2^) and 2.8 g cm^-3^ for 36 μm (10 mg cm^-2^). The mass and thicknesses of the films were measured with a microbalance and SEM, respectively. The mass loading of 1 and 3 μm-thick film is about 0.4 and 1.1 mg cm^-2^ respectively. Considering intercalated Mn ions did not provide extra capacitance, the mass of Mn-MXene-N film was measured after eliminating the cations during CV pre-cycles.

***Ex-situ* XPS and XAS measurement.** Mn-MXene-N film served as the working electrode, over capacitive activated carbon was used as the counter electrode, Ag/AgCl in 3 M KCl as the reference electrode, and 3 M H_2_SO_4_ as the electrolyte. The cell was subjected to 200 CV cycles at a scan rate of 50 mV s^−1^ in the potential range of −0.5 to 0.4 V to fully activate the material. Then the films were held at various potentials for 0.5 h and were immediately preserved in liquid nitrogen after leaving the electrolyte. The XPS and XAS spectra of all films obtained at each potential were collected in one test within 24 hours.

***In-situ* EIS measurement.** A similar three-electrode configuration with as-prepared Mn-TC-2h-N film served as the working electrode was used for *in situ* EIS test. Firstly, a CV test at the scan rate of 20 mV s^-1^ was conducted for 1 cycle, then EIS test with an amplitude of 5 mV, from 100 mHz to 100 kHz, is directly done for the film. Afterward, CV and EIS were performed alternately as shown in Fig. S32. Since EIS test with a small amplitude has little effect on the water intercalation into Mn-MXene-N, thus corresponding EIS test could be regarded as *in situ* characterization.


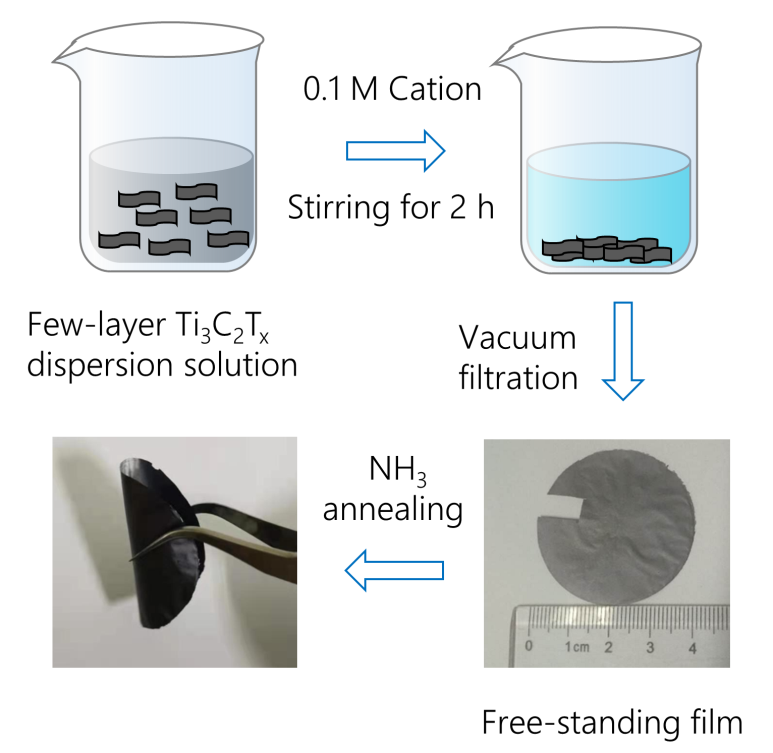


**Fig. S1.** Illustration of electrostatic assembly with redox metal cation and following ammonia treatment. The thickness of the filtrated film could be adjusted by the volume of few-layer Ti_3_C_2_T*_x_* dispersion solution. The diameter of the obtained film is 4 cm.


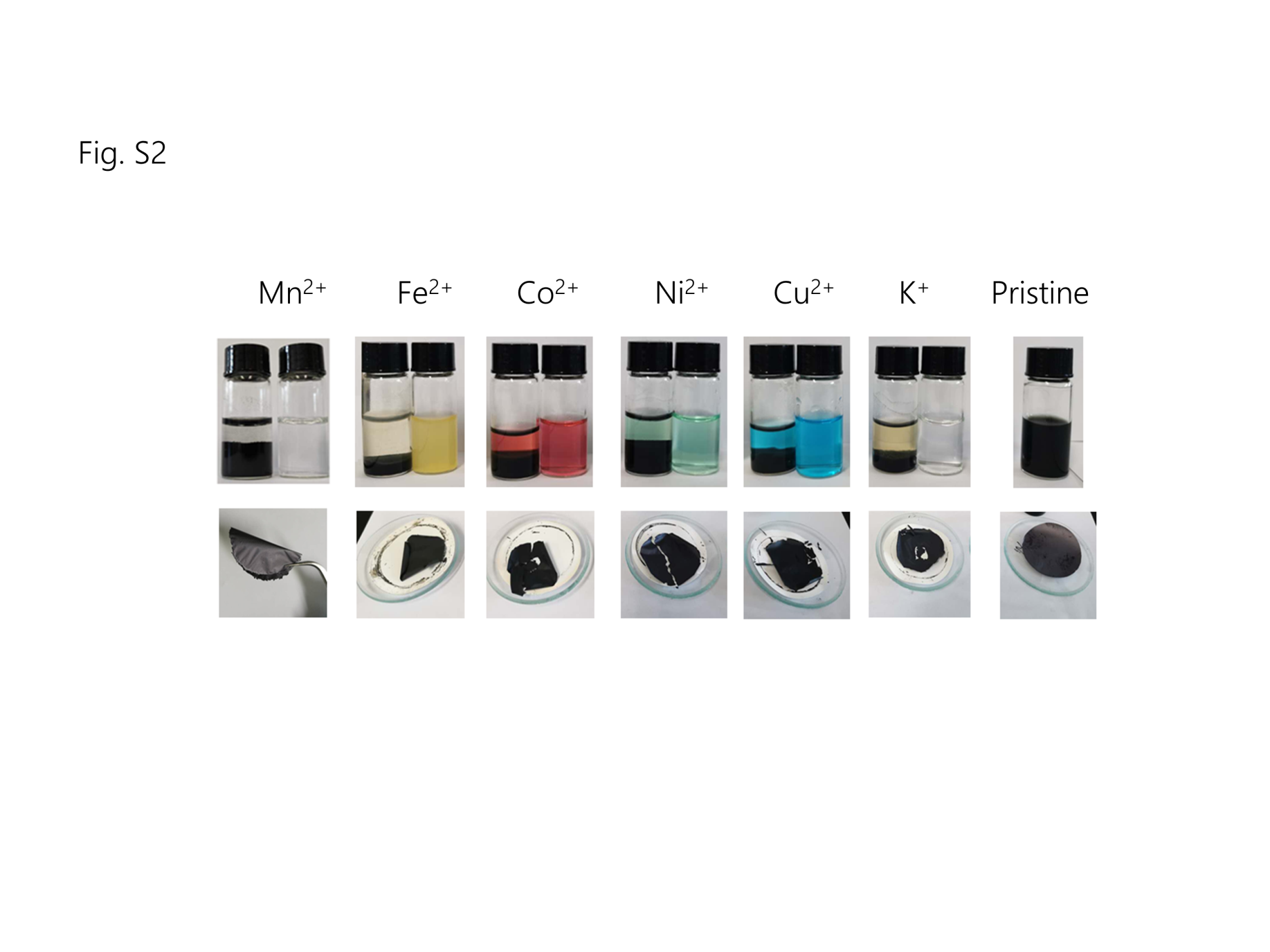


**Fig. S2.** Optical pictures of cations intercalated Ti_3_C_2_T*_x_* and the corresponding filtrated films. The vials with clear solutions contained an equal amount of cationic salt or KOH. Except for Mn^2+^ intercalated Ti_3_C_2_T*_x_* films, other films through redox cations intercalation was fragile and less conductive compared to pristine Ti_3_C_2_T*_x_* film. These results indicated that Mn^2+^ could modulate the surface chemistry of Ti_3_C_2_T*_x_* in a more controllable way, without destroying the structure, hence Mn^2+^ intercalated Ti_3_C_2_T*_x_* was used for further modifications.


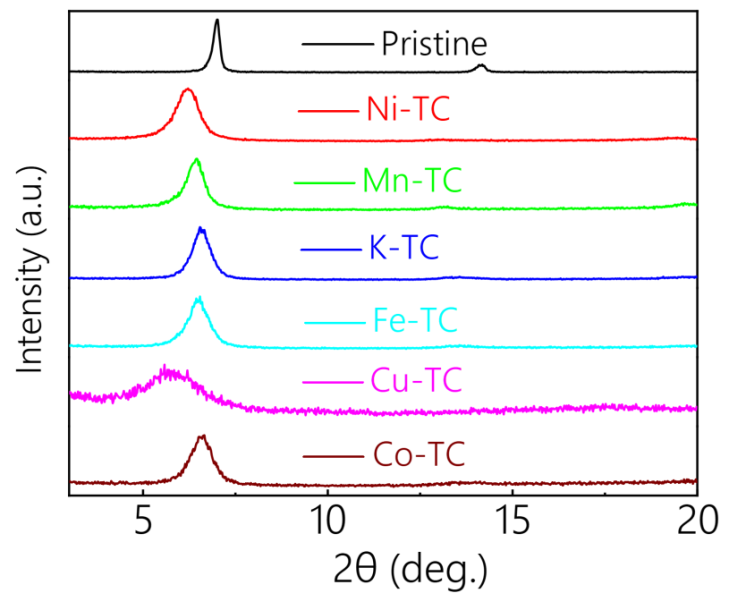


**Fig. S3.** XRD patterns of pristine and different cations intercalated Ti_3_C_2_T*_x_*. These patterns were collected from the corresponding air-dried films. Similar to *ex-situ* XRD results, the air-dried films also indicated that cation intercalation could enlarge the interlayer spacing and prevent restacking. Besides the peak shift of (002) peaks, there were intensity change of higher order (00*l*) peaks, which was a result of intercalated species and enlarged interlayer spacing.

**Table S1.** Area resistance (Ω cm^-2^) of cation intercalated films. Cation intercalation will reduce the electronic conductivity of MXene films to some extent.

| **film** | **pristine** | **Mn** | **Fe** | **Co** | **Ni** | **Cu** | **K** |
| --- | --- | --- | --- | --- | --- | --- | --- |
| **Area resistance** | **0.27** | **0.32** | **1.7** | **2.8×10^2^** | **1.4** | **1.9×10^3^** | **3.0×10^5^** |


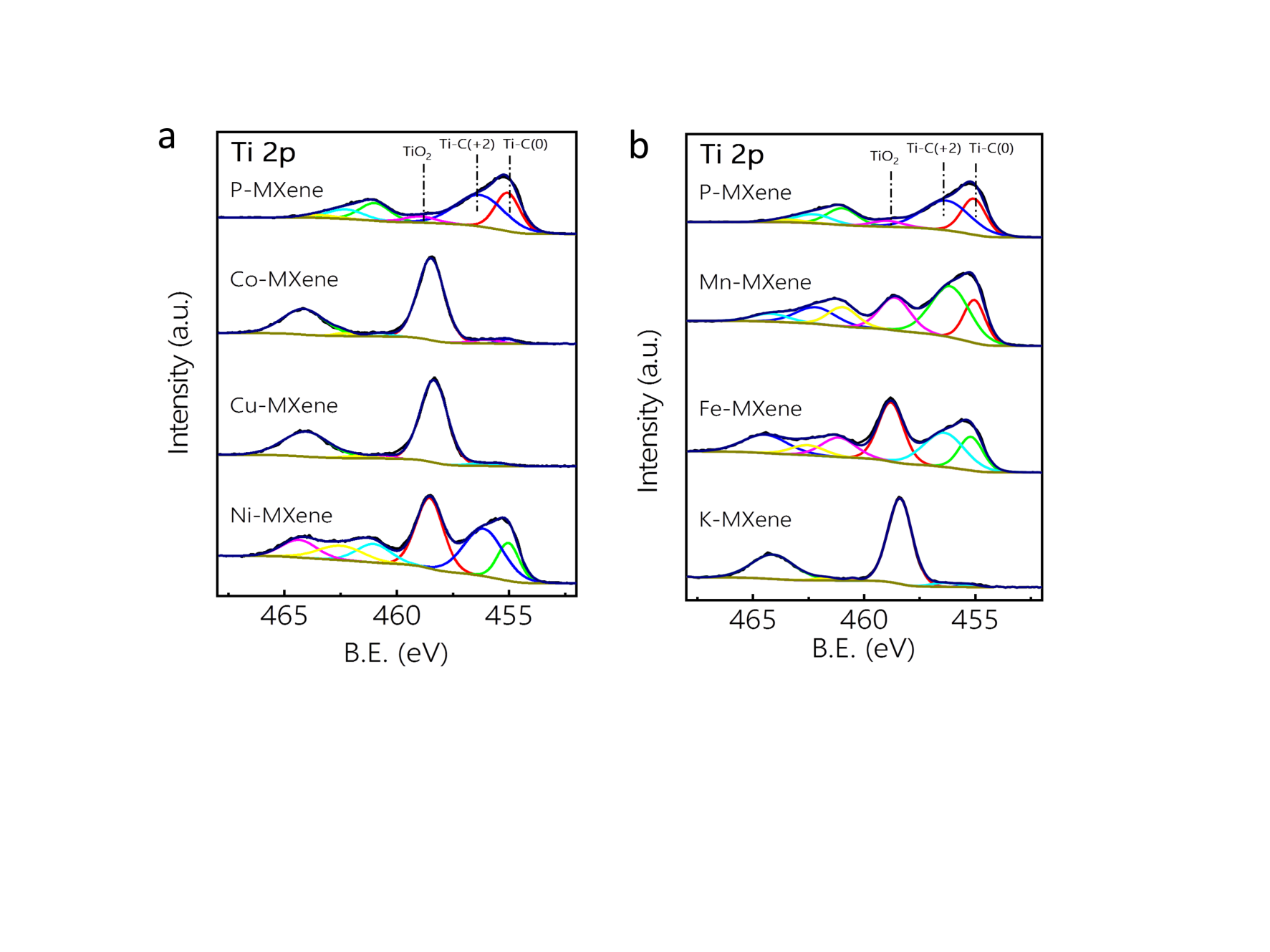


**Fig. S4.** Ti 2p XPS spectra of cation intercalated MXene. P-MXene is shown for comparison. The peaks at 455, 456.2 and 458.6 eV are assigned to the Ti-C (0 or inner Ti) (2p3/2) and Ti-C (+2 or outer Ti) (2p3/2) and TiO_2_ (2p3/2) bonds, respectively. Besides, the peaks at 460.3 eV, 461.8 and 464.1 eV are assigned to the corresponding Ti (2p1/2) states^13^. When different cations were intercalated, the peak intensity of Ti-C was reduced with that of TiO_2_ increased. When the peak of Ti-C vanished, the carbide core of Ti_3_C_2_ MXene was also destroyed, leading to severe structural damage. From XPS, we could find that Co, Cu, K (KOH, alkaline) resulted in over oxidized MXene (TiO_2_), while Mn, Fe and Ni led to partially oxidized MXene. These results were highly consistent with the area resistance results. We finally chose Mn on account of its controllable experimental procedures, high conductivity and flexibility among all obtained films.


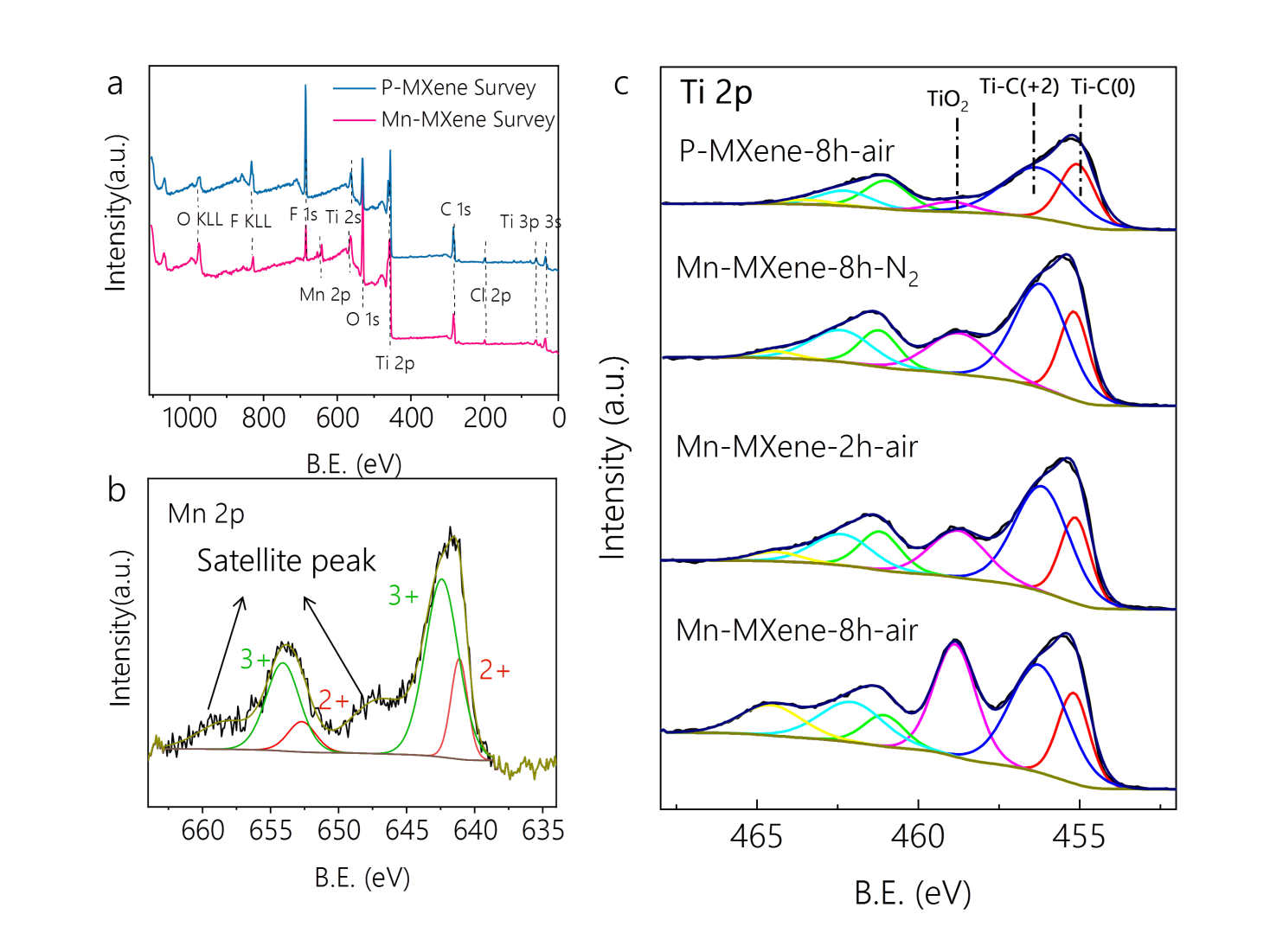


**Fig. S5. a**, XPS survey spectra of P-MXene and Mn-MXene. **b,** Mn 2p XPS spectra of Mn-MXene. **c,** XPS spectra of P-MXene and Mn-MXene under air or N_2_ atmosphere and stirred for different time.

In **Fig. S5a-b**, Mn 2p only appeared in Mn-MXene, indicating that Mn ions were successfully intercalated into Ti_3_T_2_T_x_. Compared to P-MXene, the relative intensity of F was reduced with O increased in Mn-MXene, suggesting terminal -F was substituted by -O/-OH during cation intercalation. The appearance of Mn (3+) indicates that Mn^2+^ was oxidized and the oxidant is supposed to be atmospheric oxygen^14^. The oxidation mechanism was further investigated here through XPS. From **Fig. S5c**, we found that P-MXene exposed to air for 8 h (P-MXene-8h-air) was nearly not oxidized while Mn-MXene exposed to air for 8 h (Mn-MXene-8h-air) was partially oxidized. Thus, the oxidation of atmospheric oxygen alone has little effect on surface chemistry. When Mn-MXene was purged in N_2_ for 8 h (Mn-MXene-8h-N_2_), the extent of oxidation was reduced (but still partially oxidized since O_2_ could not be thoroughly removed by purging in N_2_). In other words, Mn-MXene-8h-N_2_ has a similar oxidation extent to Mn-MXene-2h-air. Therefore, Mn cation and oxygen modulated the surface chemistry together and oxidized Mn played a key role in this process.

**Table S2.** XPS results of P-MXene and Mn-MXene. To calculate the ratio of terminal Ti-O to Ti_3_C_2_ unit (as derived from the fits of the metal spectral regions)^13^, we firstly obtained the atomic percentage of Ti and O, then the proportion of Ti-O in total O elements was also obtained. Combining these two results, the final formula was deduced as below.

| **MXene** | **Ti 2p (at%)** | **O 1s (at%)** | **Ti-O (%)** | **-OH/H_2_O (%)** | **Formula** |
| --- | --- | --- | --- | --- | --- |
| **P-MXene** | **18.2** | **20.23** | **19.5** | **80.5** | **Ti_3_C_2_O_0.65_** |
| **Mn-MXene** | **26.6** | **22.5** | **35.7** | **64.3** | **Ti_3_C_2_O_0.91_** |

**Table S3.** X-ray fluorescence analysis of P-MXene and Mn-MXene. The formula was calculated in a similar way to Table S2.

| **MXene** | **Ti (wt%)** | **F (wt%)** | **Formula** |
| --- | --- | --- | --- |
| **P-MXene** | **37.7** | **2.7** | **Ti_3_C_2_F_0.54_** |
| **Mn-MXene** | **45.2** | **1.5** | **Ti_3_C_2_F_0.25_** |

**Table S4.** Elemental analysis of P-MXene-N and Mn-MXene-N. Mn^2+^ intercalation and its effect on surface modification doubled the content of the introduced nitrogen element. The formula was obtained in Ti_3_C_2_ unit derived from the content of carbon (assuming the Ti/C ratio to be 3:2).

| **MXene-N** | **C (wt%)** | **N (wt%)** | **Formula** |
| --- | --- | --- | --- |
| **P-MXene-N** | **9.62** | **1.04** | **Ti_3_C_2_N_0.18_** |
| **Mn-MXene-N** | **9.98** | **2.09** | **Ti_3_C_2_N_0.36_** |

**Table S5.** ICP results of the residual content of Mn in Mn-MXene-N film during CV pre-cycles at a scan rate of 50 mV s^-1^. The ICP analysis gave a Ti: Mn atomic ratio and the final result was written as molar content of Mn per Ti_3_C_2_ unit (as derived from Ti content).

| **CV cycles** | **0** | **10** | **100** |
| --- | --- | --- | --- |
| **Mn content** | **Ti_3_C_2_-Mn_0.12_** | **Ti_3_C_2_-Mn_0.07_** | **Ti_3_C_2_-Mn_0_** |


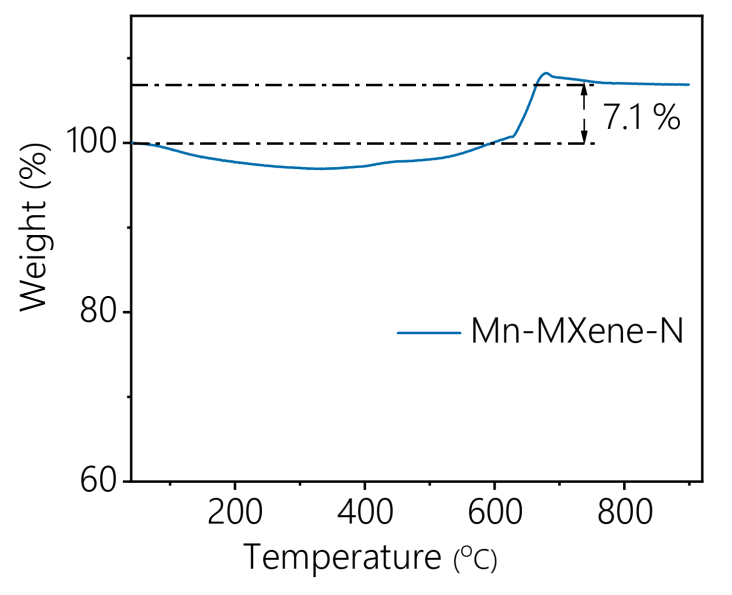


**Fig. S6.** TGA analysis of Mn-MXene-N heated under air from 40 ^o^C to 900 ^o^C. Assuming the remained sample as TiO_2_ and MnO_2_, then the exact Ti weight content in Mn-MXene-N is calculated to be 61% (Mn 3%) . Combining with carbon content in Table S3, the exact atom ratio of Ti to C was calculated to be 3: 1.97, quite close to 3: 2.


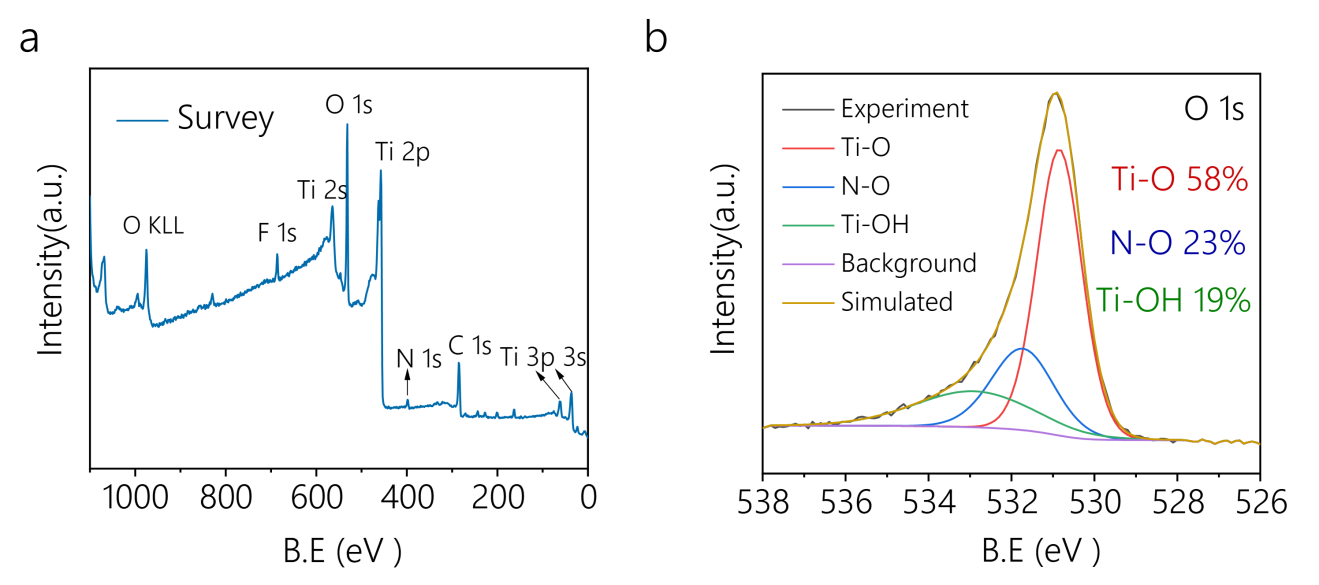


**Fig. S7. a**, XPS survey spectra and **b,** O 1s XPS spectra of Mn-MXene-N. The proportions of different components of O 1s were determined to be 58% (Ti-O), 23% (N-O) and 19% (Ti-OH), respectively. Assuming the total terminal numbers of MXenes as 2 per Ti_3_C_2_ unit, then the formula of Mn-MXene-N would be Mn_0.12_ - Ti_3_C_1.89_ -F_0.25_O_x_(OH)_1.4-x_N_0.32_. By combining the results of XPS spectra, the x is calculated to be 1.0, hence the final formula is determined.


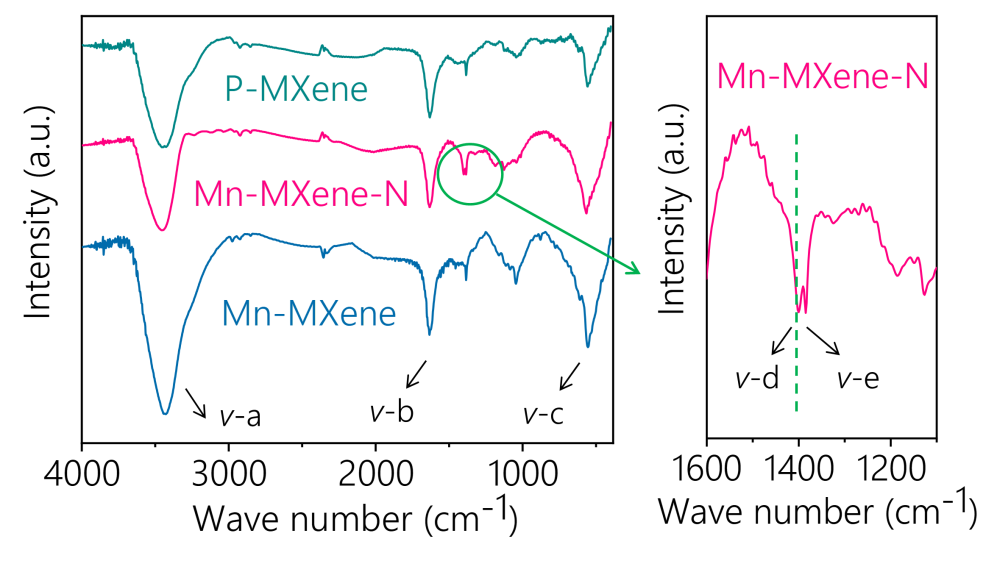


**Fig. S8.** The FTIR spectra of the indicated samples and the picture on the right is the enlarged part of Mn-MXene-N in the range of 1200-1600 cm^-1^. The peak intensities of surface -OH (3300-3500, 1620 cm^-1^) and Ti-O or Ti-O-Ti (400 – 800 cm^-1^) do not change a lot in various treatment processes, suggesting no obvious damage occurred to Ti_3_C_2_T*_x_* structure. Different vibrations assignment: *v*-a (3300-3500 cm^-1^) is the hydrogen bond of OH stretching vibration and *v*-b (1620 cm^-1^) is the deformation vibration of –OH^15^. *v*-c (400-800 cm^-1^) is the stretching vibration of Ti-O or Ti-O-Ti bridge^16^. *v*-d (1400 cm^-1^) is N=O stretching vibration^17^. *v*-e (1386 cm^-1^) is in-plane bending vibration of –OH^18^.

**Supplementary discussion 1.** There are disputes on the chemical states of doped nitrogen in Ti_3_C_2_ MXene. Lu, C. et al. ^19^ pointed out the existing form of the nitrogen dopants in previous works was still in controversy, and they suggested that the three peaks observed at 396.0, 399.7, and 401.9 eV in XPS of N 1s should be assigned to Ti-N, Ti-NH_2_ and Ti-O-NH_2_ respectively. We first adopted their identification on the chemical states of nitrogen. However, we conducted IR (Fig. S8) and found the existence of N=O stretching vibration. This could not be interpreted by Ti-O-NH_2_. Hence, we hypothesized it was Ti-N=O. When studying the origin of increased capacitance of Mn-MXene-N, we further conducted *ex situ* XPS (Fig. 4a) and found the transformation of two kinds of nitrogen state. At the same time, *in situ* XRD (Fig. 4b) also observed a gradually changed interlayer space, which is probably due to protonation of Ti-N=O (similar situations happened in Ti-O of P-MXene). This situation is chemically impossible on Ti-NH_2_ or Ti-O-NH_2_. And we also suspect its stability in 3M H_2_SO_4_ electrolytes. Finally, we proposed that nitrogen should be in the form of Ti-N-O (401.6) or Ti-N-OH (399.7). We agree that NH_3_ should be initially absorbed or substituted on the surface of MXene as suggested by Lu, C. et al. ^19^. But maybe high temperature or CV pre-cycles resulted in the transformation of nitrogen species, probably like rearrangement reaction in organic chemistry. Moreover, an article reporting that NH_3_ annealed TiO_2_ (similar surface chemistry to Ti_3_C_2_ MXene with Ti-O/OH) finally yielded Ti-NO species on the surface during photocatalysis convinces us the transformation of Ti-NH_2_ or Ti-O-NH_2_ is possible^20^.


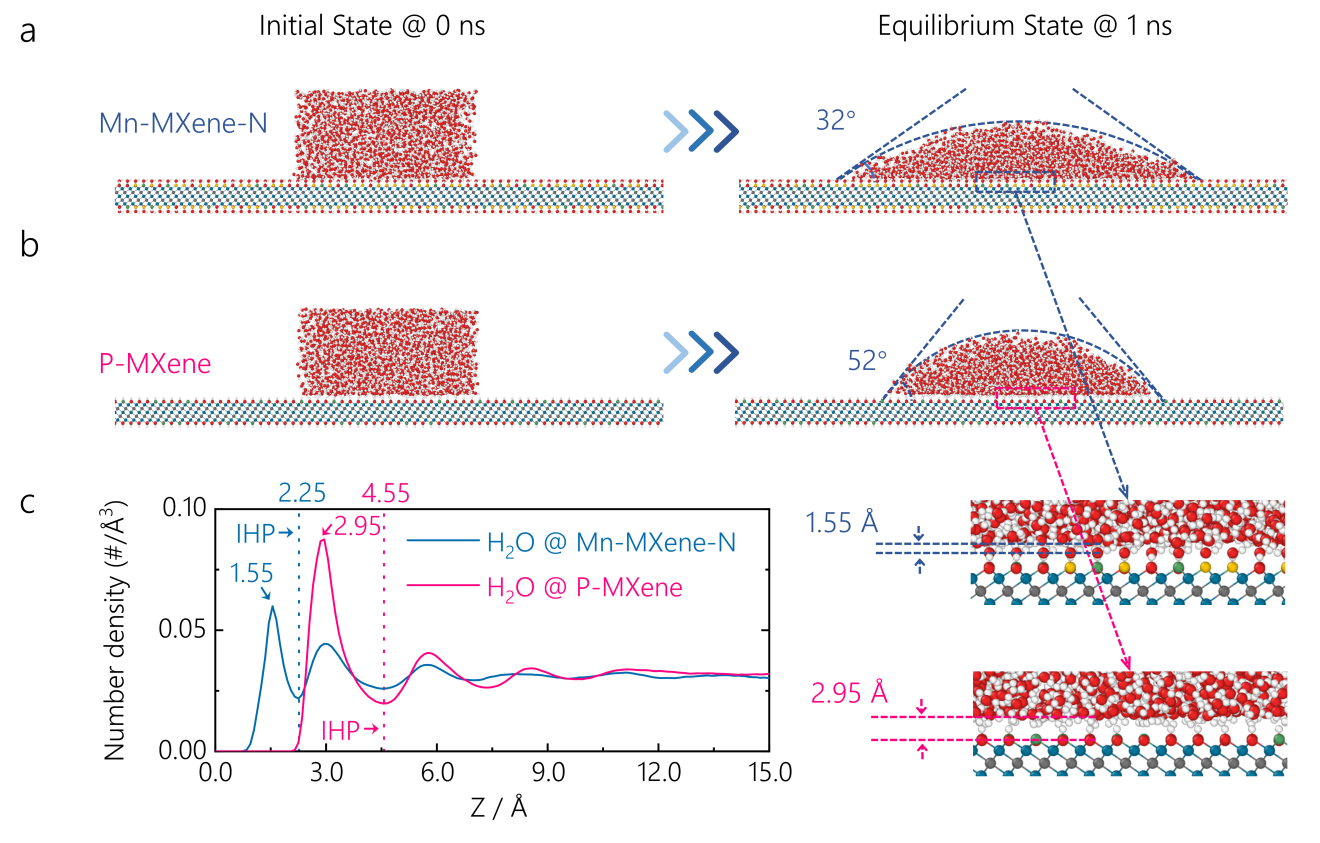


**Fig. S9.** Snapshot of **a,** Mn-MXene-N and **b,** P-MXene surface water droplet hydrophilic process calculated through MD simulation. At initial state (t = 0 ns), a radius = 30 Å and height = 30 Å cylindrical water drop is freely placed on the surface of Mn-MXene-N and P-MXene, after a 1 ns long simulation run, both systems reach equilibrium state. The contact angles of both equilibrium state water droplet are marked with guidelines. It could be found that the Mn-MXene-N system shows a better hydrophilic with the smaller contact angle, which is consistent with the experimental results. **c,** the number density profile of water molecules along the direction perpendicular to the Mn-MXene-N and P-MXene surface (*Z* axis). *Z* = 0 indicates the position of the outermost atom layer of Mn-MXene-N and P-MXene surface. Under the effect of better hydrophilic and stronger surface polarity, the first water peak of Mn-MXene-N system migrates to a closer position at 1.55 Å (compared with 2.95 Å of P-MXene), and the thickness of the inner Helmholtz plane (IHP) is reduced to 2.25 Å (compare with 4.55 Å of P-MXene), denoting a closer surface contact of aqueous electrolyte and the Mn-MXene-N surface, which is beneficial for the double electrode layer energy storage.


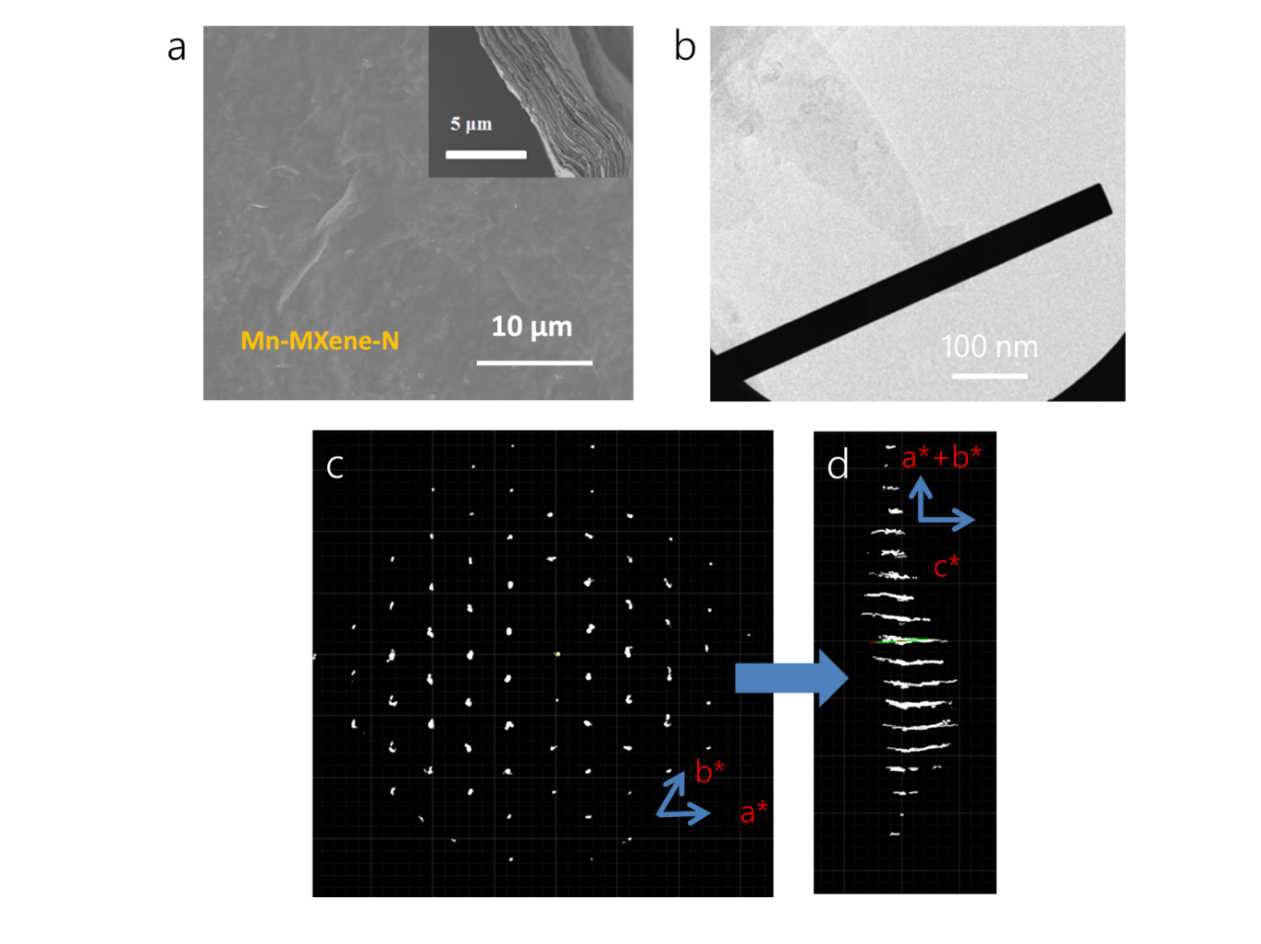


**Fig. S10. a,** SEM images of surface and cross-section morphology of Mn-MXene-N. The morphology of Mn-MXene-N before and after 10000 CV cylces remain quite similar, indicating the structure and morphology of Mn-MXene-N remain undamaged after cycling for a long time. Reconstructed 3D reciprocal lattice of Mn-MXene-N from RED data: **b,** The cryo-TEM image of Mn-MXene-N flakes. **c,** The reciprocal lattice along *c**, perpendicular to the MXene layer. **d,** The reciprocal lattice parallel to the MXene layer, which is obtained from rotating **c** by 90°.


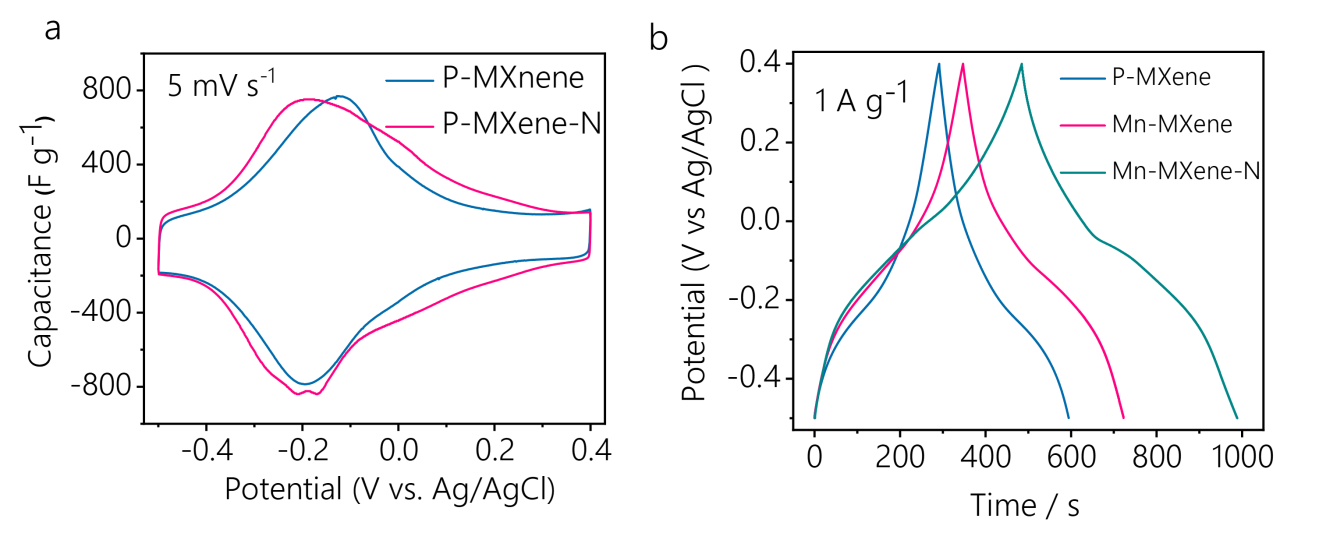


**Fig. S11. a,** Cyclic voltammograms of P-MXene and its nitrogen-doped form (P-MXene-N) at a scan rate of 5 mV s^−1^ in the potential range from -0.5 V to 0.4 V versus Ag/AgCl**. b,** Galvanostatic charging/discharging profiles of the indicated samples collected at 1A g^-1^. The coulombic efficiency of Mn-MXene-N under 1 A/g and 10 A/g was 96.4% and 99.5%, respectively.





**Fig. S12.** Inverse of stored charge (1/*q*) versus the square root of the scan rate (*v*^1/2^) derived from CV curves of the P-MXene, Mn-MXene and Mn-MXene-N film electrodes, with *v* from 5 to 100 mV s^−1^. The extrapolation of *q* to *v* = 0 in **Fig. S12** gives the total charge (*q_T_*) while extrapolation of *q* to *v* → ∞ in Fig. 3c can obtain the non-diffusion-controlled charge (*q_n_*). The deviation from linear relationship above 100 mV/s in Fig. 3c is probably due to the limited ion transport.


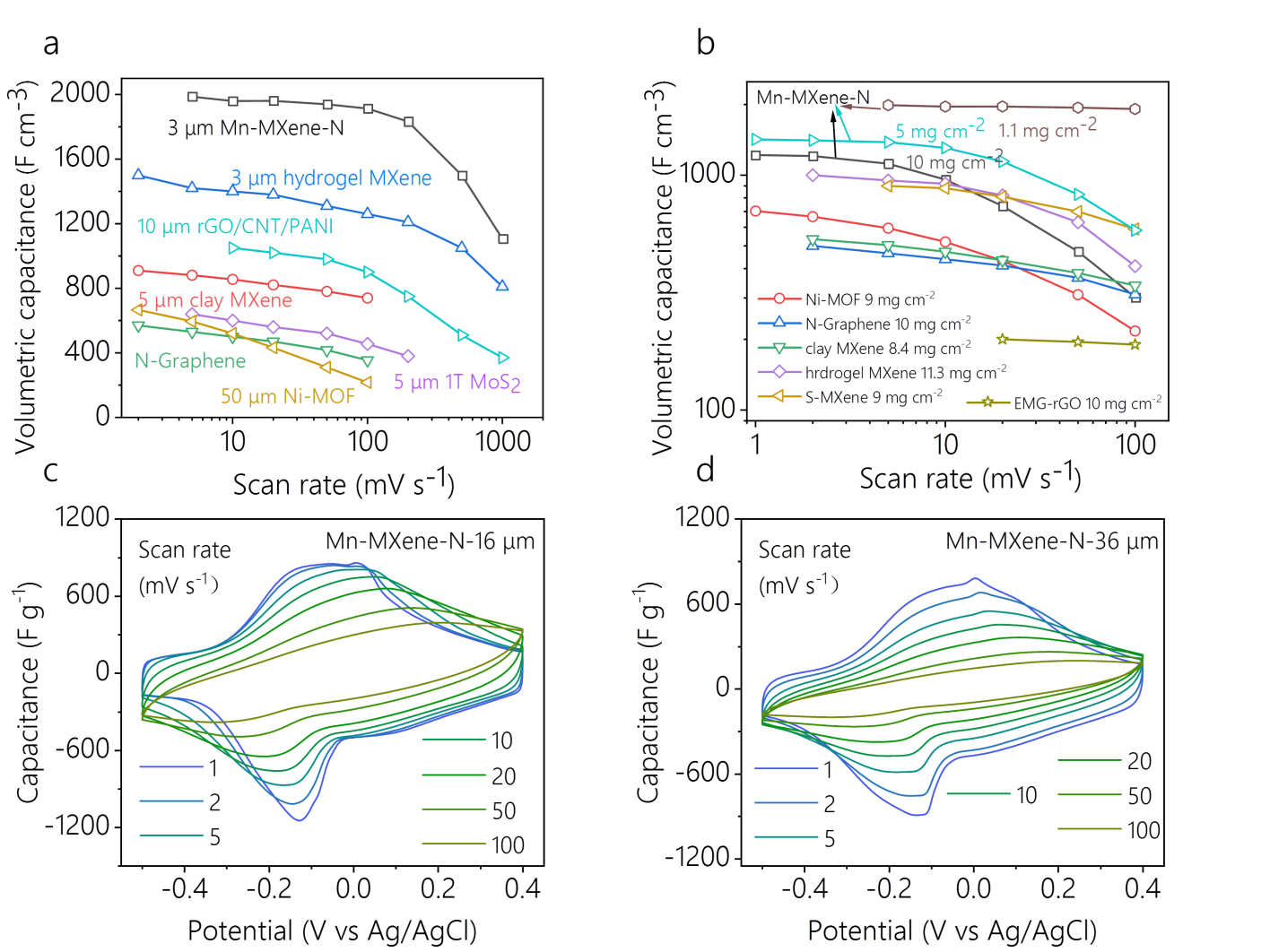


**Fig. S13. a,** Comparison of the volumetric capacitance at different scan rates of 3μm Mn-MXene-N with the performance of other materials. **b,** Comparison of the volumetric capacitance Mn-MXene-N with mass loading from 1.1 mg cm^-2^ to 10 mg cm^-2^ and other materials with high mass loading. These materials include hydrogel MXene^21^, MXene clay^22^, rGO/CNT/PANI^23^, 1T MoS_2_^24^, N-Graphene (nitrogen doped)^25^, Ni-MOF^26^, S-MXene^27^ and EMG-rGO^28^. N-Graphene and Ni-MOF did not report their performance of thin films, thus we prepared thick Mn-MXene-N films (16 μm and 36 μm) for fair comparison. As for N-graphene and Ni-MOF with mass loading about 10 mg cm^-2^, our material with similar loading shows better capacitance at the scan rate from 1-50 mV s^−1^ and comparable at 100 mV s^−1^. Cyclic voltammograms of **c,** 16 μm (5 mg cm^-2^) and **d,** 36 μm (10 mg cm^-2^) Mn-MXene-N at scan rates from 1 mV s^−1^ to 100 mV s^−1^. Even at a high mass loading up to 10 mg cm^-2^, Mn-MXene-N still delivers a comparable volumetric capacitance with the state-of-art electrode materials. The unsatisfactory performance at high scan rates is mainly due to sluggish ion transportation when using the film assembly method through vacuum filtration, which might be addressed through electrode morphology design.

**Supplementary discussion 2.** There are some articles^29-31^ reporting a higher volumetric capacitance than this work. However, we found that these articles doubled their specific capacitance by mistake. For example, Que et al.^29^ reported that UN-Ti_3_C_2_@5 mV s^-1^ delivered a capacitance of ~900 F g^-1^ or 2800 F cm^-3^. But we calculated the specific capacitance through integral their CV curve and the result is actually ~ 460 F g^-1^ or 1400 F cm^-3^. In their supporting information, they found that the specific capacitance of their best material is about 460 F/g at 0.5 A g^-1^, a similar discharge rate to 5 mV s^-1^ (Fig. S14b UN-Ti­_3_C_2_@0.5 A g^-1^), consistent with our calculation. Fig. S14c is the CV Curve @ 5 mV s^-1^ of Mn-MXene-N in our work and Fig. S14d is the overlap of Fig. S14a and c. Obviously, our material has a higher integral area with a wider potential range (0.9 V vs 0.7 V), indicating a superior specific capacitance in our work. Similar mistakes could be found in these papers^29-32^. Thus, we excluded these articles when comparing the volumetric capacitance.


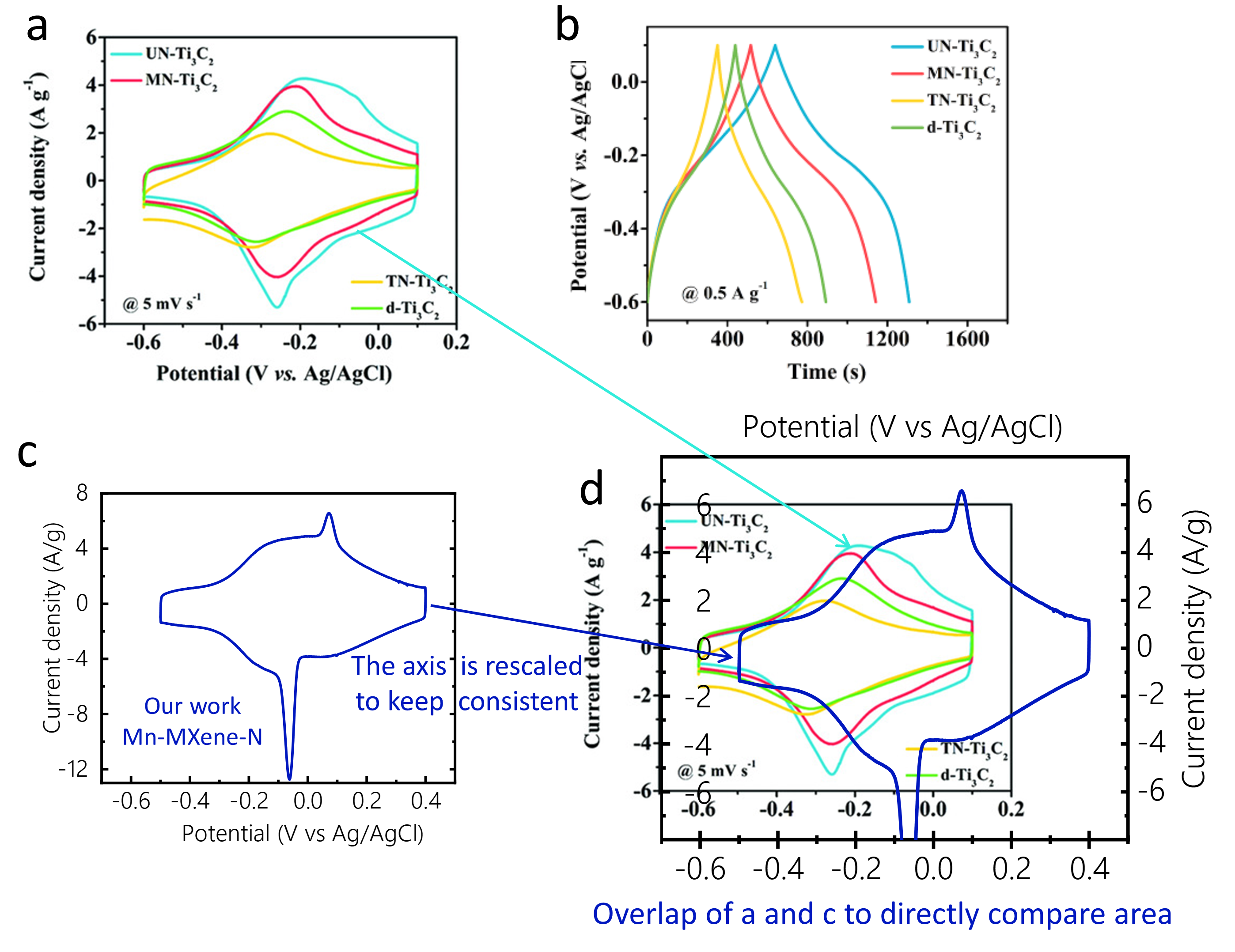


**Fig. S14**. **a,** CV curves of the freestanding d-Ti_3_C_2_, TN-Ti_3_C_2_, MN-Ti_3_C_2_, and UN-Ti_3_C_2_ film electrodes in 3 M H_2_SO_4_ solution at scan rate of 5 mV s^-1^, taken from reference 29; **b,** GCD curves of the indicated films at 0.5 A g^-1^, taken from reference 29; **c,** CV curves of the freestanding Mn-MXene-N film electrode in our work; **d,** the overlap of CV profiles from **a,** and **c**.


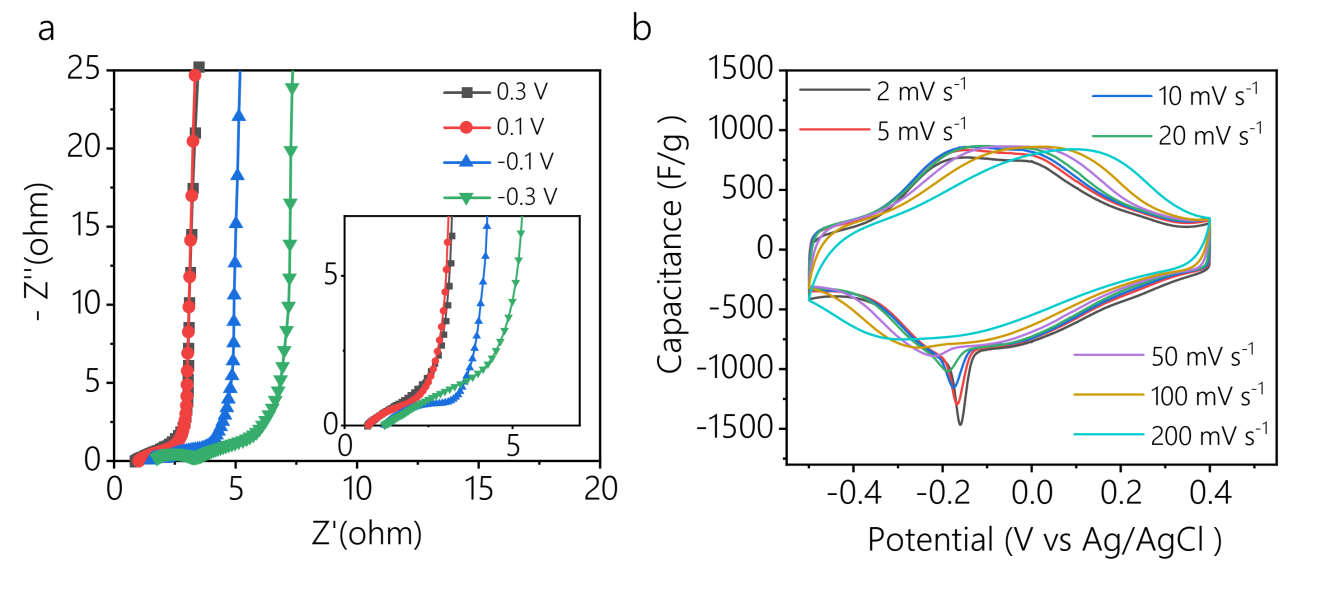


**Fig. S15. a,** Electrochemical impedance spectroscopy data collected at different potentials for 5-um-thick Mn-TC-N film (mass loading of 1.9 mg cm^-2^). The inset shows the high-frequency range. **b,** Cyclic voltammetry data collected at scan rates from 2 to 200 mV s^-1^ of 5-um-thick Mn-MXene-N. To investigate the electrochemical behavior of Mn-MXene-N, EIS experiments at different potentials are conducted in a similar way as previous study^21^ (Fig. S15a). The Nyquist plots at potentials of 0.3 V and 0.1 V tend to overlap and are characterized by a low ion transport resistance and a sharp rise of the imaginary component of impedance at low frequencies (almost vertical), demonstrating the capacitive behavior of the electrode. As can be seen from both EIS (Fig. S15a) and CV profiles collected at different scan rates (Fig. S15b), this potential region corresponds to a relatively low capacitance originating from non-diffusion limited processes. By contrast, an obvious increase of the 45-degree linear part (related to ion transport resistance) and a less steep slope of the Nyquist plot in the low-frequency range at -0.1 V and -0.3 V are observed. This correlates with the appearance of redox peaks in the CVs (Fig. 3b) associated with a pseudocapacitive mechanism. Thus, the EIS results suggest the capacitive behavior of Mn-MXene-N above 0.1 V, hence the increased current density in this potential range is mainly derived from the ELD-like mechanism.


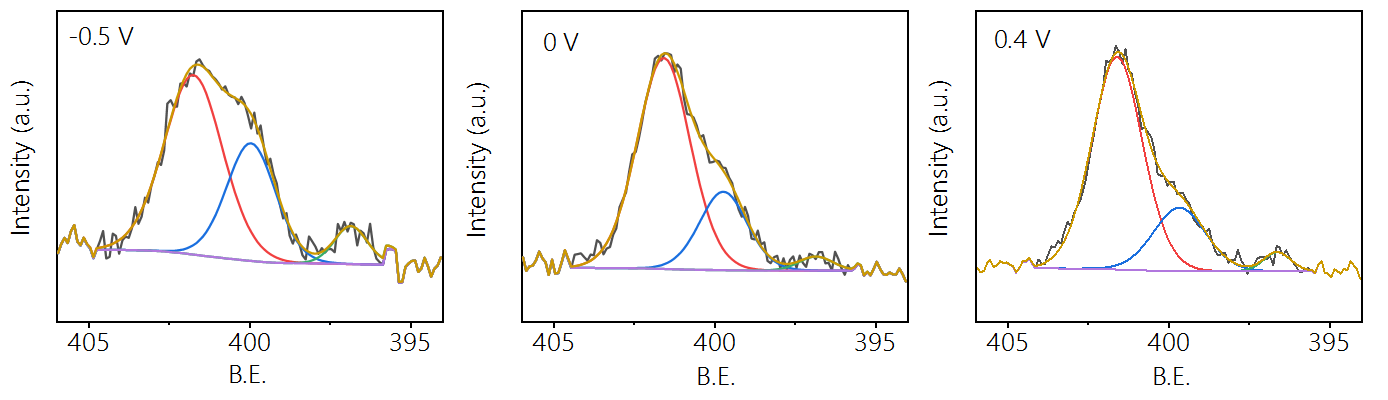


**Fig. S16.** *Ex-situ* XPS spectra of N 1s for Mn-MXene-N after holding at different potentials. The three distinct positions are referred to as Ti-N-O (401.6 eV), Ti-N-OH (399.7 eV) and Ti-N (396.7 eV) respectively. The proportion of Ti-N-O is reduced with the applied potential decreased. Ti-N only takes a minor part (about 3-7%). Its effect on surface chemistry and interlayer water are negligible, thus was not fitted in Fig. 4a for better semi-quantitative analysis.


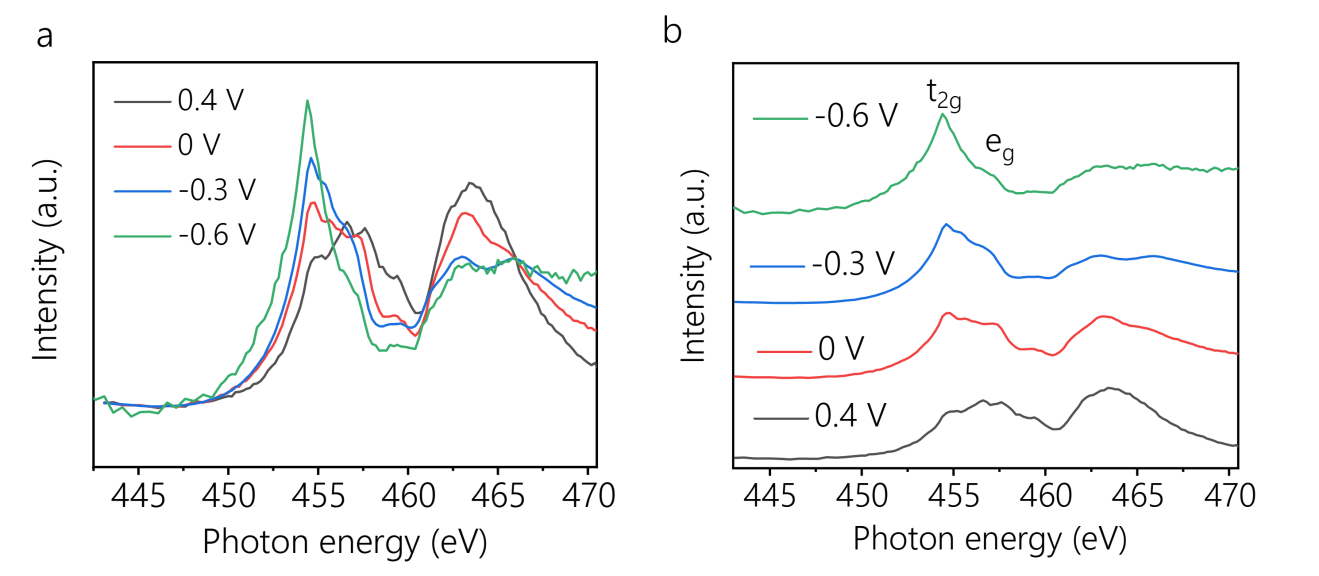


**Fig. S17. a,** The comparing and **b,** the stacking *ex-situ* Ti L-edge XAS spectra of Mn-MXene-N collected after holding in the applied potentials vs Ag/AgCl. XAS was recorded in TFY mode in vacuum. The photon energy range from 450-460 eV is related to the excitation of Ti 2P_3/2_ (L_3_-edge) core levels to an unoccupied Ti 3d state. Owing to the ligand field, the Ti L_3_-edge is split into two sub peaks corresponding to electronic states with t_2g_ and e_g_ symmetries. Since XAS is highly sensitive to the Ti chemical environment, we could trace the evolution of the surface terminals when the applied potential changed. The increase in the e_g_ component is supposed to be the evidence of a higher oxidation state of Ti^33^.


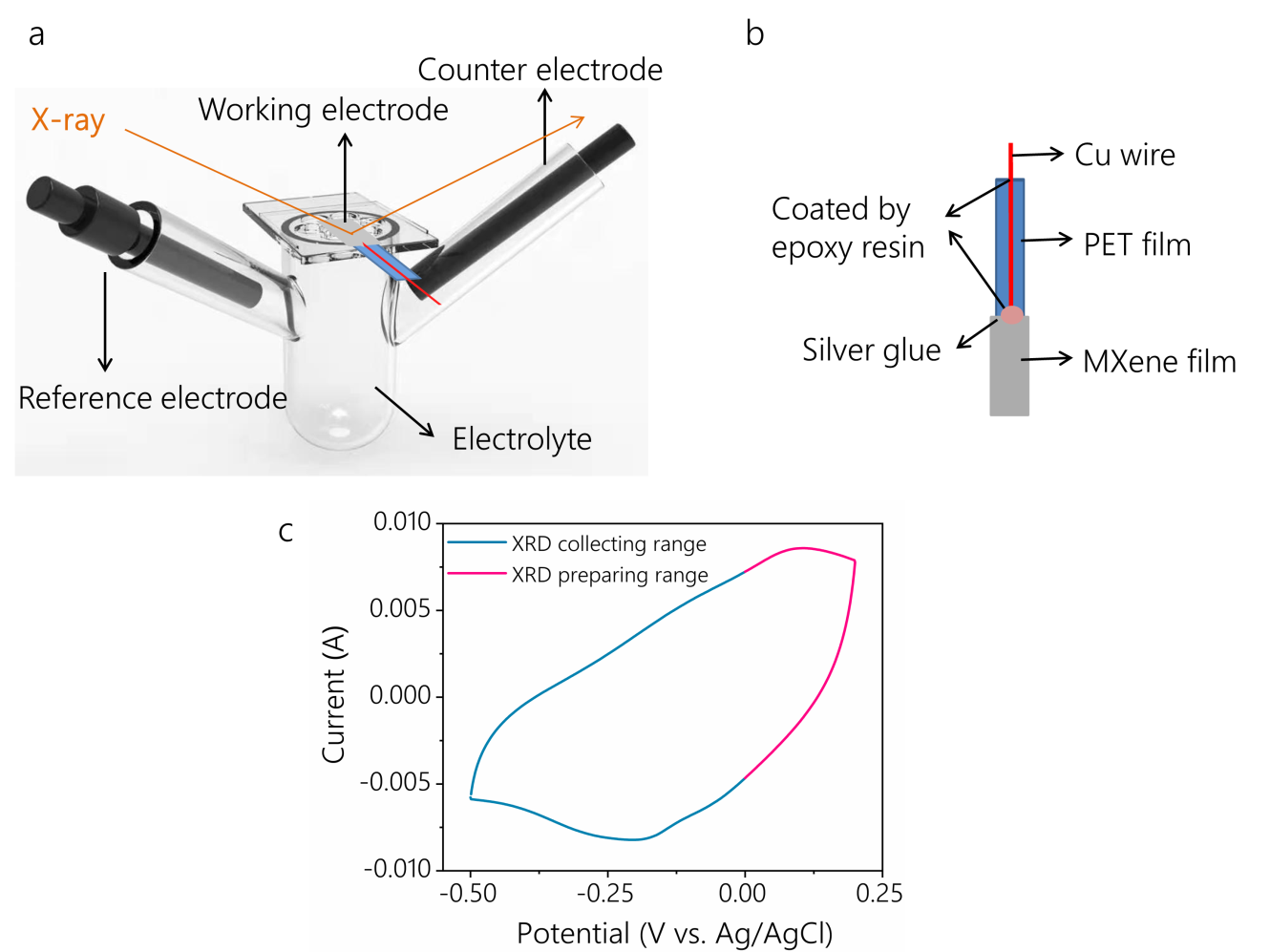


**Fig. S18. a,** The schematic of modified *in situ* XRD cell, in which free-standing MXene film served as working electrode, carbon rod as counter electrode, Ag/AgCl in 3M KCl as reference electrode and 3M H_2_SO_4_ as electrolyte. **b,** the schematic of the free-standing working electrode. **c,** Illustration of *in situ* activation XRD. In one complete CV cycle, only the grey part collects the corresponding XRD data.

Considering most commercial *in-situ* XRD cells are mainly for batteries, which are usually used for two-electrode systems and not resistant to acid, we designed this simple cell to trace the interlayer spacing change of Mn-MXene-N during cyclic voltammetry test. This modified cell was used to investigate two problems, one is how the interlayer spacing changes during charging/discharging, and the other is the water intercalation phenomenon during CV pre-cycles.

In *in-situ* activation XRD, we used batch operation to continuously collect dozens of XRD patterns. For each CV cycle at 20 mV s^−1^, it will take 70 s to accomplish charge and discharge. When XRD machine performs a batch operation, there will be interval time from the end of the last XRD to the start of the next XRD collecting operation (it was nearly 20 s ). Then, we set the scanning time of XRD collecting process to be about 50 s. We ensured that the time of each XRD data collecting and the interval time of machine preparation were added precisely to 70 s. For each cycle of XRD collecting, CV pre-cycle was started from 0 V and scanned to negative polarity (0 V to -0.5V) and followed by scanning to possitive polarity (-0.5V to 0.2V), while no data in potential range between 0.2 V and 0 V will be collected (Fig. S18c). In *in-situ* activation XRD experiment, we used a narrower potential range, free-standing working electrode without charge collector (Fig.S18 b) and a larger disk of sample for high quality XRD patterns (0.28 cm^2^ in a standard test but 2 cm^2^ for in situ activation XRD). These factors will lead to the deviation of the shape of CV curve (Fig. 5a), but will not affect the investigation on the role of water intercalation.


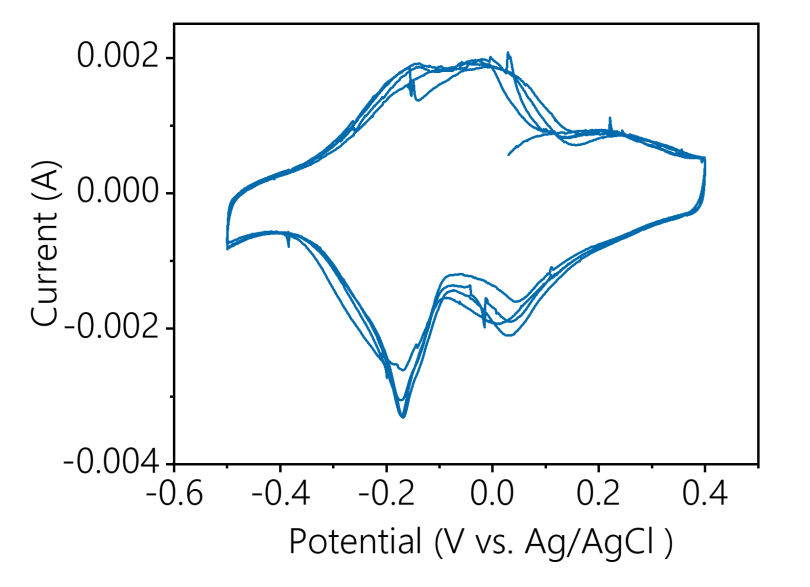


**Fig. S19.** Cyclic voltammogram of Mn-MXene-N at 0.5 mV s^−1^. In this experiment, Mn-MXene-N was firstly activated thoroughly with at least 100 CV pre-cycles. Then, we collected this CV profile during *in situ* XRD experiment to probe how interlayer spacing changes during charging and discharging. Due to water volatilization, we added H_2_SO_4_ electrolyte once during the long-time *in-situ* XRD experiment.


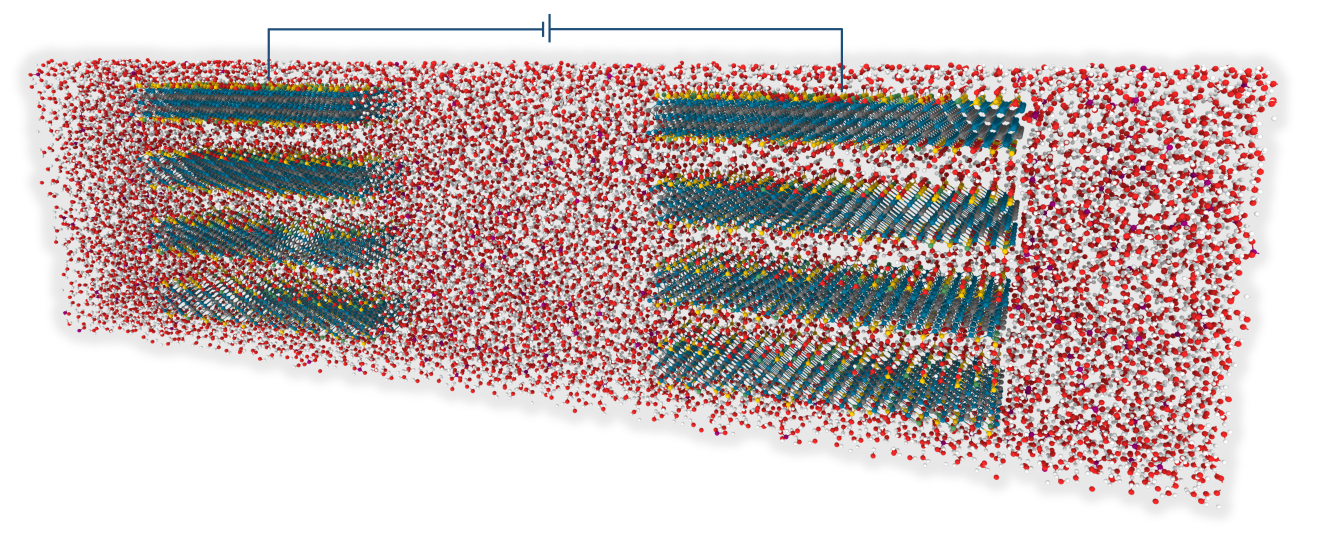


**Fig. S20.** Schematic of the MD simualtion model of MXene-based supercapacitors. The simulation system consists of two indentical four-layer Mn-MXene-N or P-MXene electrodes connected with 3 M H_2_SO_4_ reservoirs.





**Fig. S21.** The intercalation/deintercalation numbers of H_2_O, H_3_O^+^ and SO_4_^2-^ (HSO_4_^2-^) during charging/discharging process. The intercalated number of H_2_O is tenth that of H_3_O^+^ (Fig.R2), hence it is reasonable to mainly attribute water intercalation to the interlayer spacing change.


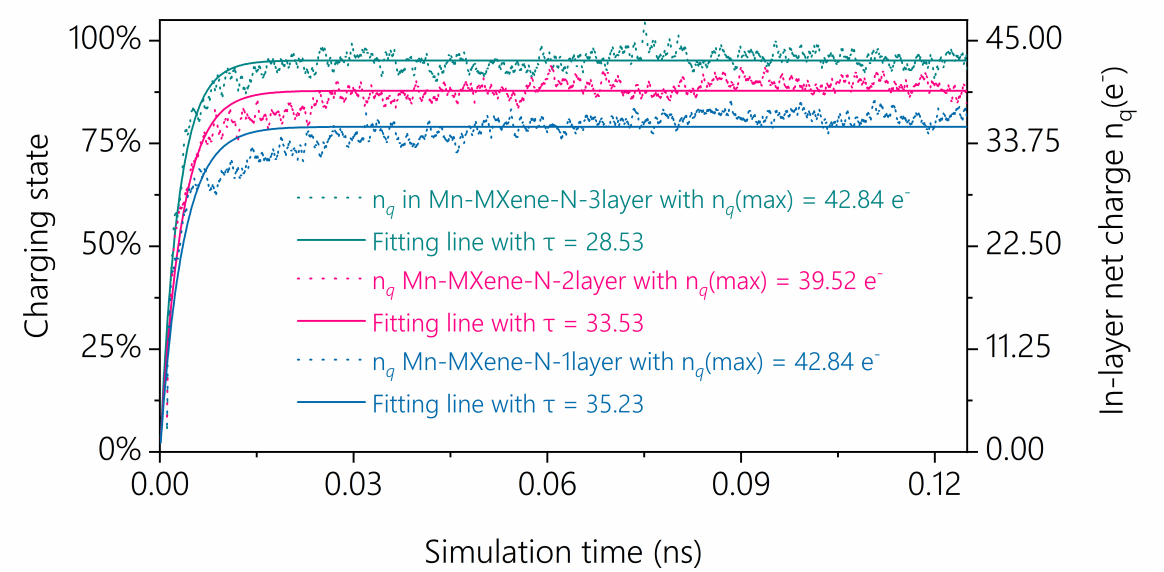


**Fig. S22.** Time evolution of net charge n*_q_* between the layers after a cell voltage applied between two electrodes, displayed by the negative electrode, for (a) Mn-MXene-N-3layer, (b) Mn-MXene-N-2layer, (c) Mn-MXene-N-1ayer. The fitting lines represent the curves by fitting MD-results into the RC transmission line equivalent circuit model, and the time constant τ for each charging process is calculated and annotated, respectively.


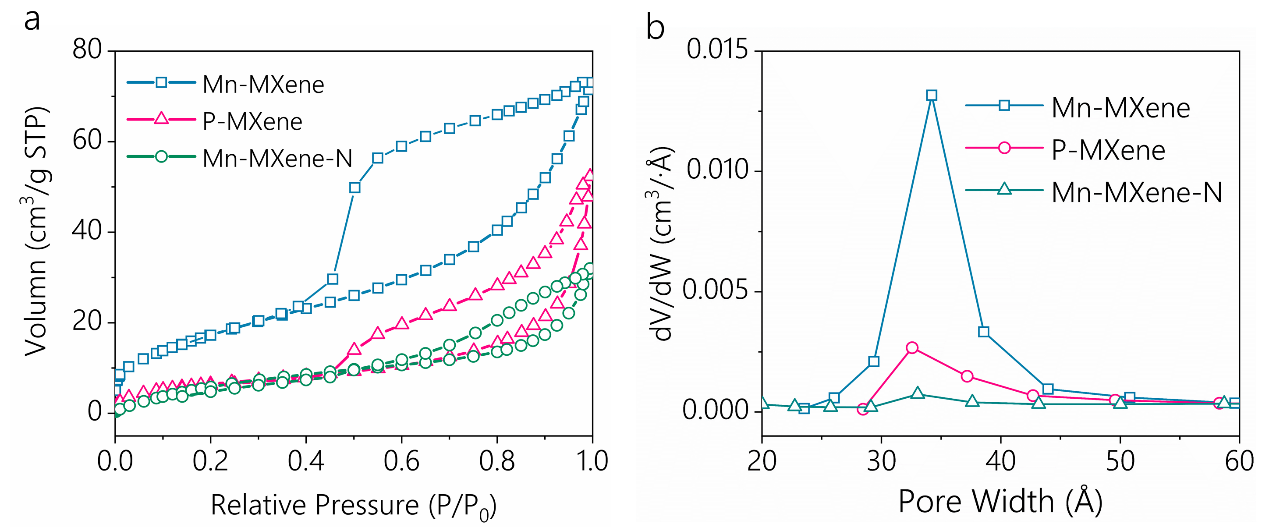


**Fig. S23. a,** N_2_ adsorption-desorption isotherms of P-MXene, Mn-MXene and Mn-MXene-N. **b,** their corresponding pore-size distributions. The specific surface areas (SSAs) are measured to be 23.7, 65.1, and 28.1 m^2^ g^-1^ for P-MXene, Mn-MXene and Mn-MXene-N, respectively. Mn-MXene has the highest SSA due to the swelling effect of cation intercalation. The SSA of Mn-MXene-N is smaller than Mn-MXene, which is probably a result of annealing. Though there is difference between the SSAs of three samples, all the SSAs are small, consistent with the compact morphology of MXene films. However, we indeed observed considerable capacitive contribution despite low SSA results. To explain this, we speculate that relatively small slit-like channels in MXene are difficult for N_2_ to access but enough for proton (mainly through interlayer water), thus, the electrochemical surface area might be actually very large and similar for three samples. Under the circumstances, the width of interlayer void will take dominant effect in EDL-like capacitance, as discussed in our manuscript.


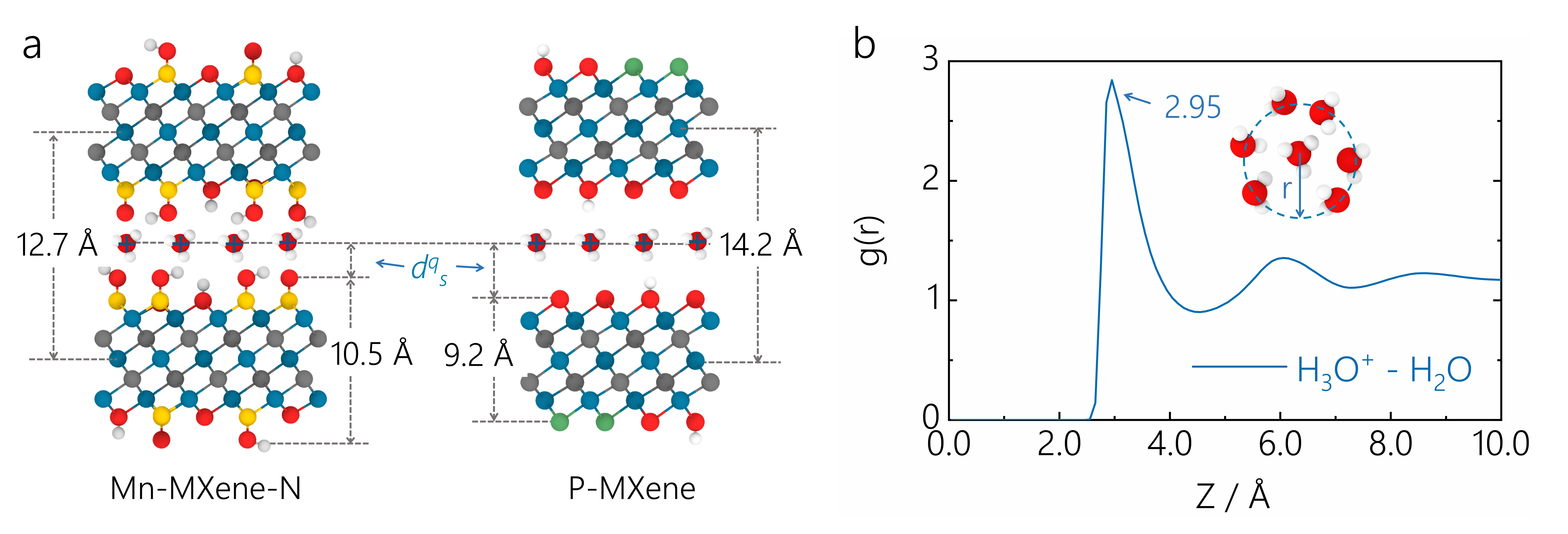


**Fig. S24.** **a**, Calculation of the charge separation distance *d^q^_s_* between the center of charge and the surface of the electrode at the 1-layer water inside case. *d^q^_s-_*_Mn-MXene-N_ =*d_space between layers_* / 2 = (12.7 -10.5) / 2 = 1.1 Å, *d^q^_s-_*_P-MXene_ = *d_space between layers_* / 2 = (14.2 - 9.2) / 2 = 2.5 Å. **b**, The radial distribution function (rdf) between H_3_O^+^ and H_2_O. We can see the peak of the first solvation shell of H_3_O^+^ *r_ss_* is 2.95 Å, this 2.95 Å radius is larger than both *d^q^_s-_*_Mn-MXene-N_ and *d^q^_s-_*_P-MXene_, indicates a solvation and de-solvation would happen at both cases during charging and discharging. The larger deviation between *r_ss_* and *d^q^_s-_*_Mn-MXene-N_ than *r_ss_* and *d^q^*_P-MXene_ denotes stronger solvation and de-solvation, the calculated coordination number *Nc*_-Mn-MXene-N_ for H_3_O^+^ decreasd to 2.4 (from *Nc* = 4.4, at bulk state) in the 1-layer Mn-MXene-N void, while the *Nc*_-P-MXene_ for H_3_O^+^ is 3.3 in 1-layer P-MXene void.


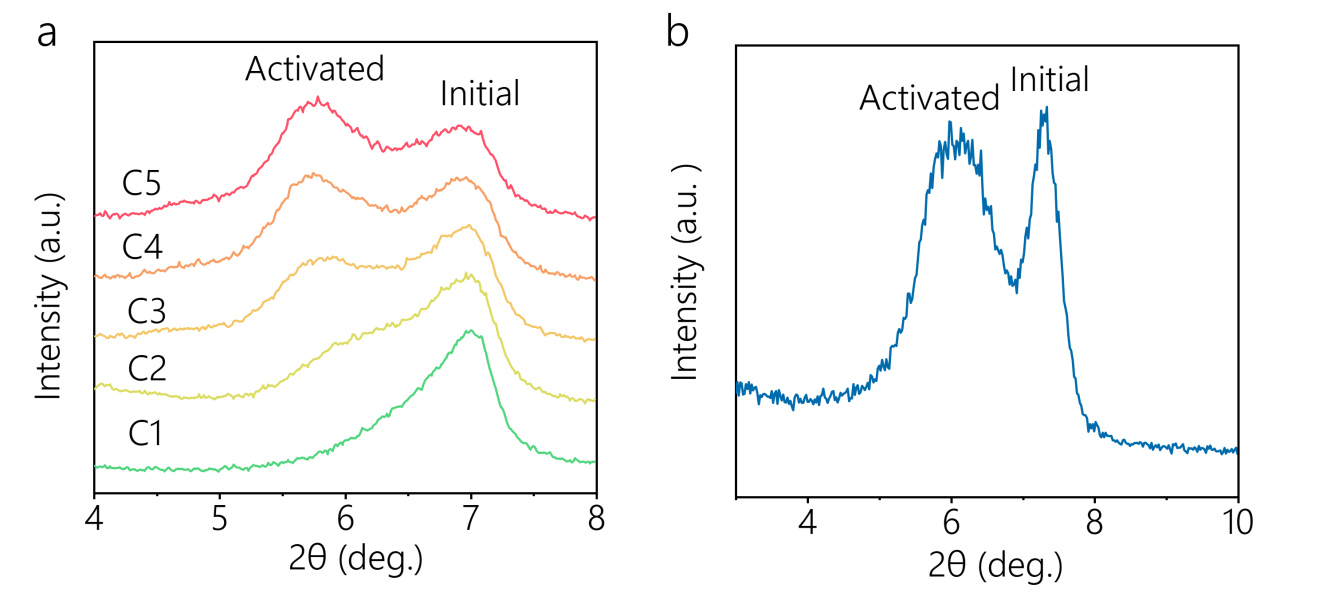


**Fig. S25. a,** *In situ* XRD patterns of Mn-MXene-N film at the first 4 CV pre-cycles. **b,** *Ex-situ* XRD pattern of Mn-MXene-N film at the first 10 CV pre-cycles.


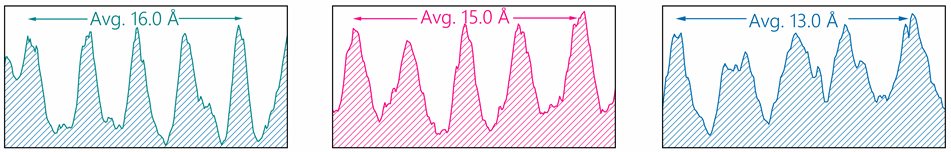


**Fig. S26.** The average interlayer spacing from cryo-HRTEM images in Fig. 5c.

**Supplementary discussion 3.** Atomically imaging the O atoms of water by TEM is challenging because of the small scattering cross section^34^. It is true that direct water image by HRTEM was reported through confining ordered water (ice) in-between monolayer graphene^35^. But this work was questioned by Zhou., *et al*^36^. Thus, there are still disputes on whether water can be visualized by HRTEM. In the case of our work, water confined by MXene is more difficult to be visualized than in graphene. Ti_3_C_2_T*_x_* MXene is at least five-atom-thick with disordered terminals and the confined water was also calculated to be disordered. Therefore, it is nearly impossible to visualize confined water with electron beam directly going through thick MXene in normal direction like the case in graphene^35^. In addition, it is also extremely hard to atomically visualize confined water with electron beam going through MXene interlayer due to the disordered water arrangement. As for other methods to directly image water, direct water imaging was usually done on the ultra-clean surface by AFM/STM^37-39^, but it is extremely difficult to directly detect nanoconfined water in-between MXenes currently.


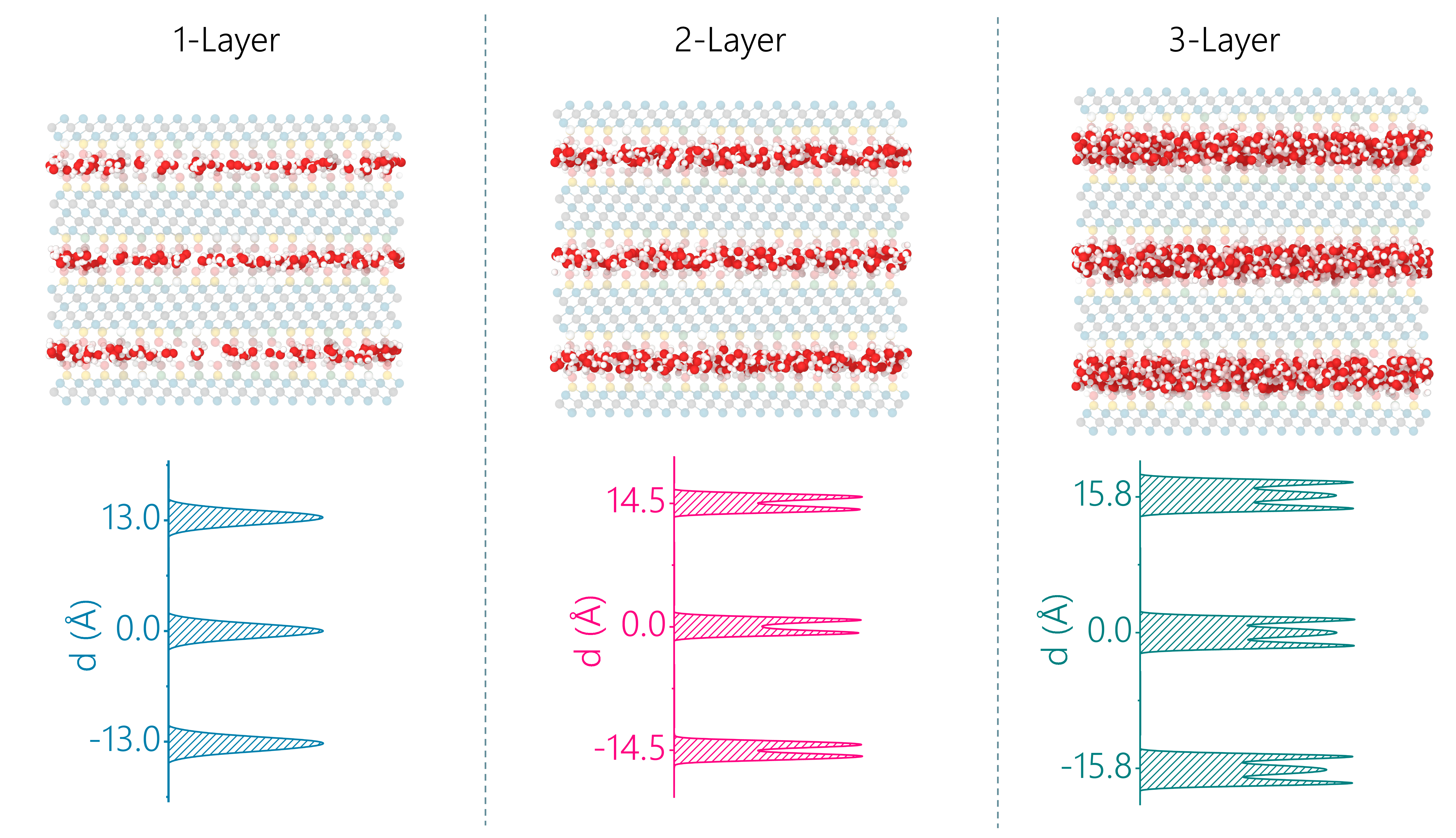


**Fig. S27.** The distribution of confined water with discrete layers under different interlayer spacing. (a) 1-layer (b) 2-layer (c) 3-layer

**Supplementary discussion 4.** In addition to the investigations on the effect of confined water on electrochemical performance, here we further discussed the nature of layered nanoconfined water. When water strongly confined in nano-slits, its macroscopic physical properties would be significantly changed^40-44^. We find that either liquid or molecular form is inaccurate to describe confined water. We tend to consider nanoconfined water in a quasi-crystalline form which is typical in a confined nano-sized void, with the proton relaxation time between free water and ice or surface-bound hydrogen (Low field ^1^H NMR in Fig. 5g). Nanoconfined water is also speculated to show nonlinear layered properties, since one-layer confined water contacts both surfaces of MXene simultaneously; when confined water is two layers, it is reasonable to assume that both layers of water interact with the surface of MXene and interact with each other; when confined water is increased to three layers, water in the middle layer would be likely to bulky water as it directly interacts with other layered water. From this point of view, we could preliminarily know three-layer nanoconfined water has properties close to bulky water.

MD simulations are also conducted here to understand the arrangement of confined water. The probability profiles, P(θ), of the dipole orientation of the interlayer water molecules of the 3-layer case at uncharged and charged states are shown below. It clearly shows that the dipole orientations of up-layer, middle-layer, down-layer water are all concentrated around 90° at uncharged state, which means all the three layers of water tend to a parallel arrangement (nearly parallel to the MXene layers). Differently, at charged state, the up-layer water and down-layer water shown a clear dipole orientation rearrangement, while the middle-layer water nearly maintains their original orientation trend, as shown in the figure below. At charged state, the near-surface layer water will rearrangements when experiencing the electrostatic force and rotates to help to screen the electrostatic field.


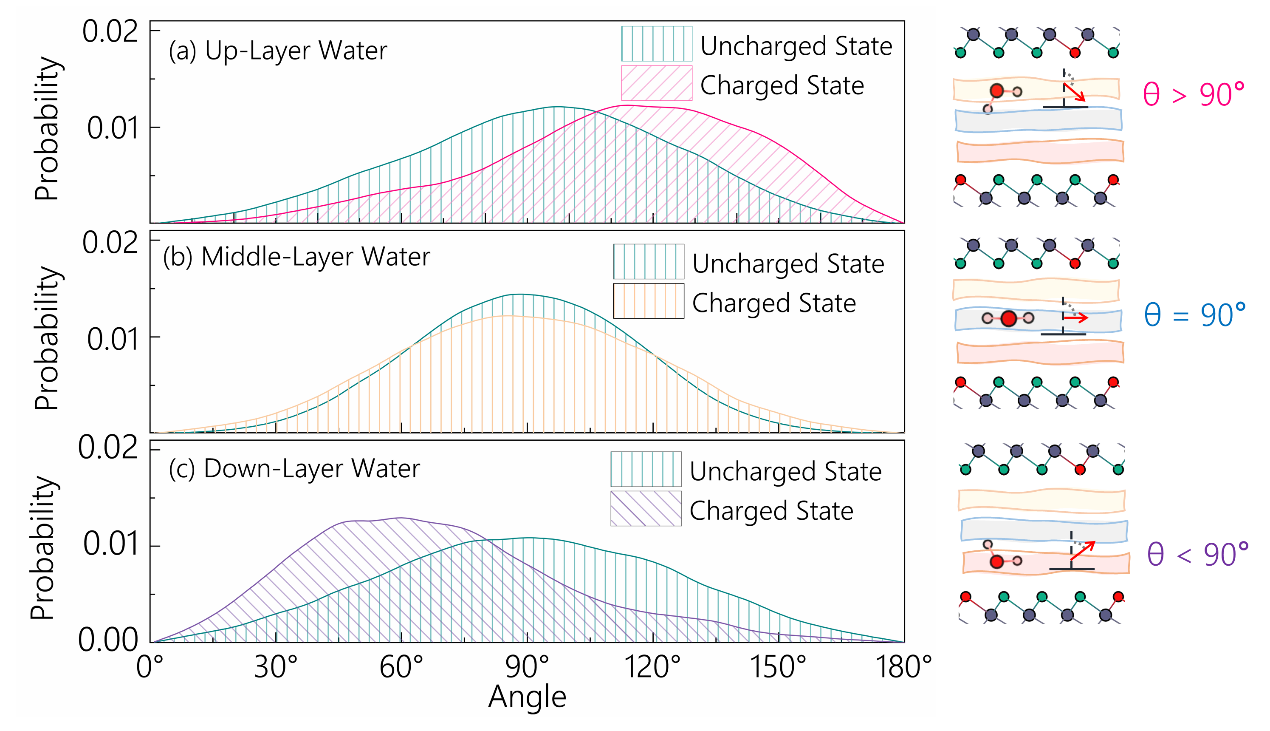


**Fig. S28.** Comparison of probability profiles of dipole orientation of up-layer **a,** middle-layer **b,** down-layer **c,** water molecules inside Mn-MXene-N layers at uncharged state and charged state. θ gives the angle between the water molecular dipole moment and the down-layer electrode surface normal. The right atomic schematic diagrams exhibit the different layer water dipole orientation tendency inside the charged Mn-MXene-N layer.

Cryo-dried experiment is further conducted to confirm the nonlinear property of three-layered water. We firstly fully activated Mn-MXene-N and charged it to -0.5 V vs Ag/AgCl. Then, we cryo-dried Mn-MXene-N under a pressure of 5 Pa at -55 ^o^C for three days. Fig. S28 shows the XRD patterns of charged and discharged Mn-MXene-N as well as the cryo-dried Mn-MXene-N. The (002) peaks of charged and discharged Mn-MXene-N were located at 5.5^o^ and 6.9^o^, corresponding to MXene with three-layer (16 Å) and one-layer water (12.8 Å), respectively. Interestingly, the (002) peak of cryo-dried Mn-MXene-N is located at 6.2^o^ with the interlayer spacing of 14.3 Å, which could be assigned to MXene with two-layer water. Considering three days are usually enough to cryo-dry MXene dispersions, we speculate that the middle-layered water might show similar behavior to free water or water in mesopores.





**Fig. S29.** the XRD patterns of indicated samples charged and discharged Mn-MXene-N as well as the cryo-dried Mn-MXene-N


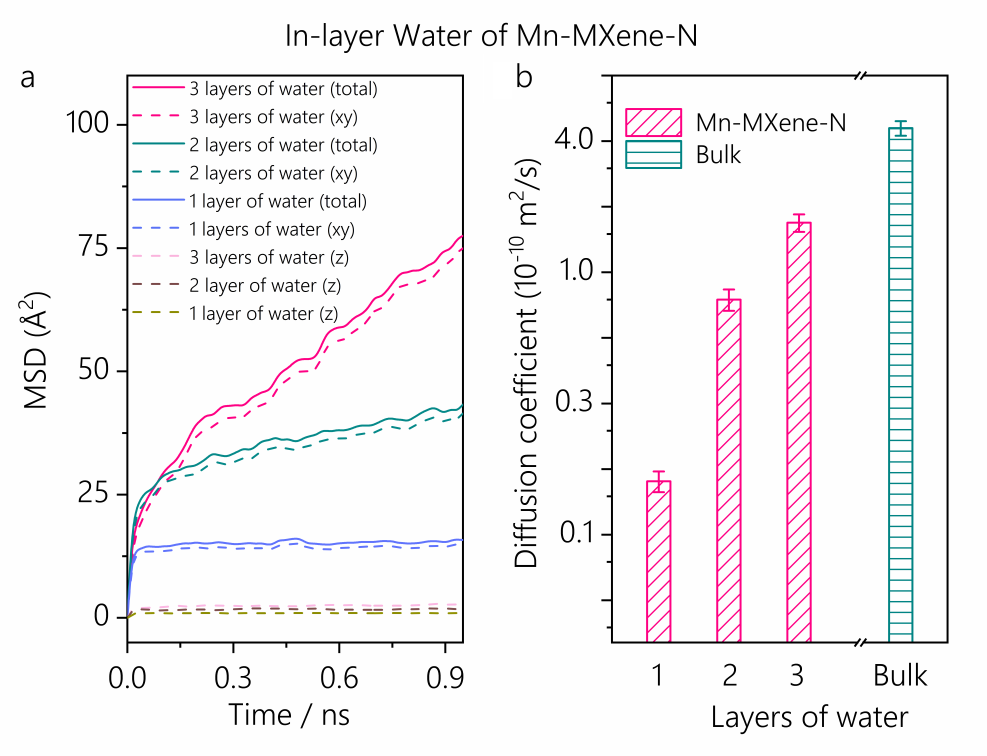


**Fig. S30.** Water mobility and proton diffusion in Mn-MXene-N confined water layers: **a,** total and decomposed mean-square displacement (MSD) of the O atom. **b,** proton diffusion coefficients for different number of water layers (Bulk water are included for comparison). Error bars represent the standard deviation of the diffusion coefficients calculations. When the interlayer space between MXene layers is empty, a larger interlayer spacing can naturally accommodate more layers of nanoconfined water. According to the MD simulations, nanoconfined water shows layer-dependent properties, and the more layers of nanoconfined water, the higher water mobility and proton diffusion coefficients.


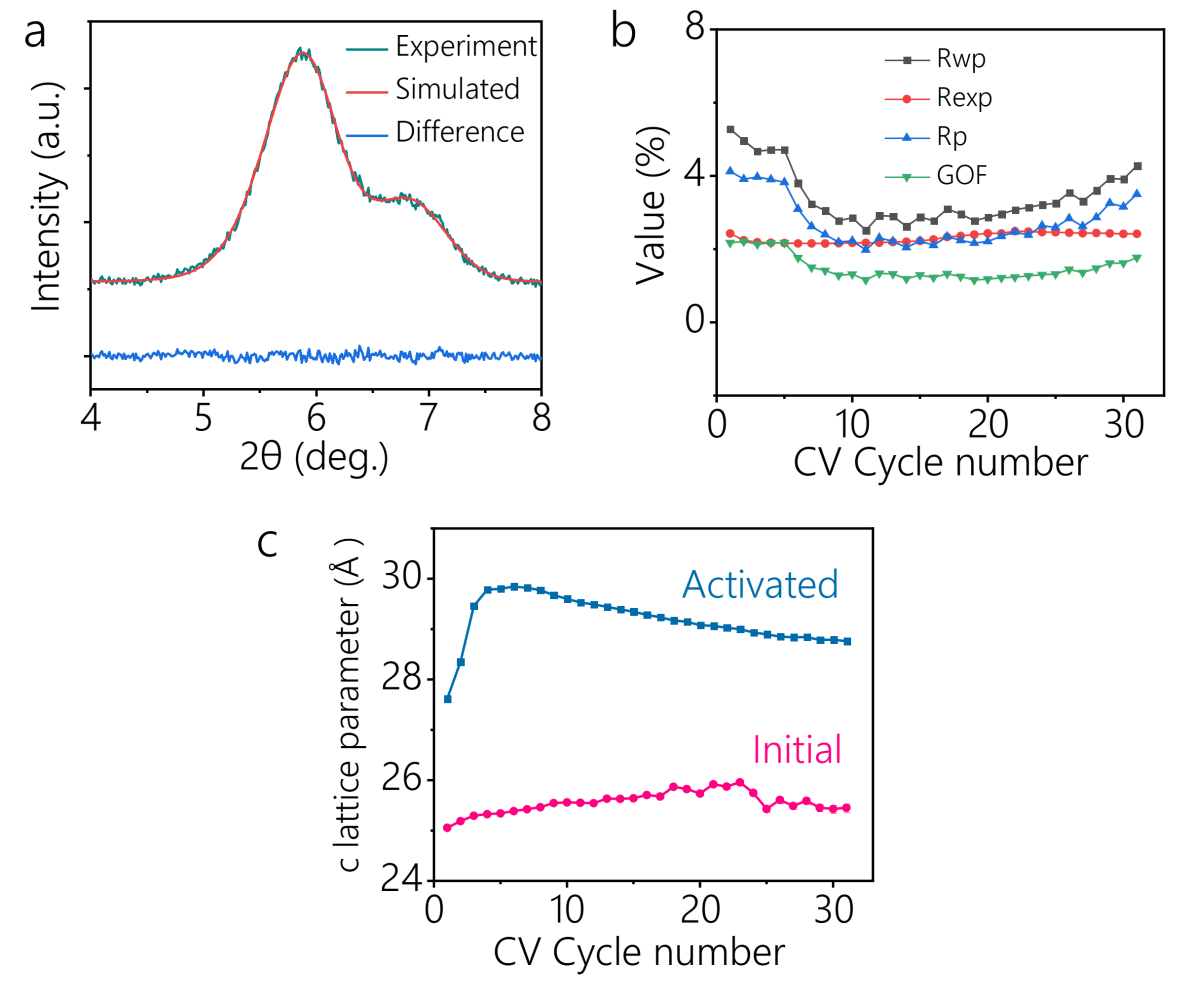


**Fig. S31.** The le bail fitting results of the in situ electrochemical XRD scans. **a,** The le bail fitting pattern of C11 is shown here for example. **b,** The agreement factors of fitting and **c,** the refined cell parameter c change of both phases for all scans. The Le bail refinement was performed against each *in situ* XRD scan to extract the cell parameter c with TOPAS 5.0. The initial cell parameters c of the initial phase and the activated phase were set to 25.24 Å and 28.07 Å respectively. Thompson-Cox-Hasting pseudo-Voigt peak shape and Chebyshev polynomial background were used. Since only 002 reflections of both phases within the data range, only peak shape parameter, background and cell parameter c was refined. The proportion of these two parts are extracted for each XRD pattern through revietld refinement by assuming the same structural factors for both.


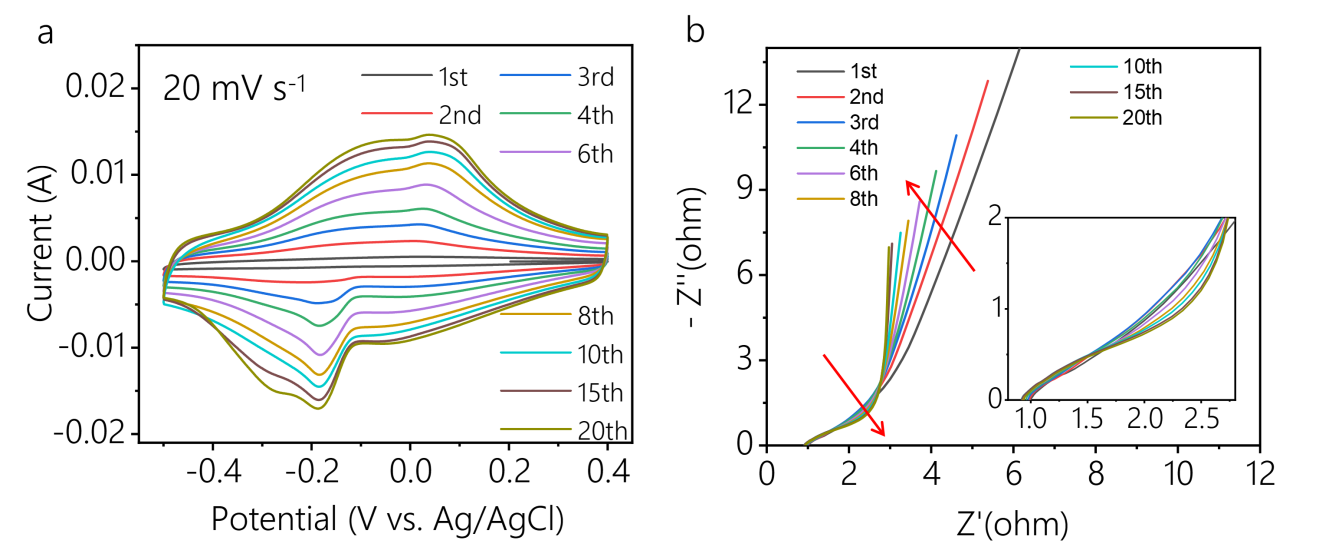


**Fig. S32.** *In situ* EIS test of Mn-MXene-N during CV pre-cycles. **a,** 20 CV pre-cycles of Mn-MXene-N at a scan rate of 20 mV s^-1^. **b,** Nyquist plots of Mn-MXene-N at -0.1 V vs Ag/AgCl after indicated electrochemical CV cycles.


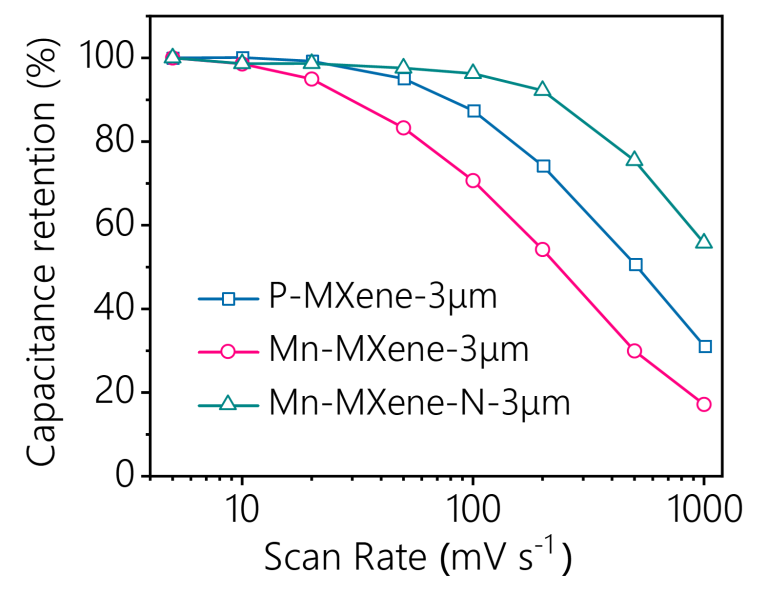


**Fig. S33.** Capacitance retention of 3-μm-thick P-MXene, Mn-MXene and Mn-MXene-N films at the scan rate from 5 to 1000 mV s^-1^.

**Reference:**

1 Alhabeb, M. *et al.* Guidelines for Synthesis and Processing of Two-Dimensional Titanium Carbide (Ti_3_C_2_T_x_ MXene). *Chem. Mater.* **29**, 7633-7644 (2017).

2 Lukatskaya, M. R. *et al.* Cation intercalation and high volumetric capacitance of two-dimensional titanium carbide. *Science* **341**, 1502-1505 (2013).

3 Zhang, W.-H. *et al.* Graphene oxide membranes with stable porous structure for ultrafast water transport. *Nat. Nanotechnol.* **16**, 337-343 (2021).

4 Zheng, L. *et al.* Robust ultraclean atomically thin membranes for atomic-resolution electron microscopy. *Nat. Commun.* **11**, 541 (2020).

5 Plimpton, S. Fast Parallel Algorithms for Short-Range Molecular Dynamics. *J. Comput. Phys.* **117**, 1-19 (1995).

6 Stukowski, A. Visualization and analysis of atomistic simulation data with OVITO–the Open Visualization Tool. *Modell. Simul. Mater. Sci. Eng.* **18**, 015012 (2009).

7 Cygan, R. T., Liang, J.-J. & Kalinichev, A. G. Molecular Models of Hydroxide, Oxyhydroxide, and Clay Phases and the Development of a General Force Field. *J. Phys. Chem. B* **108**, 1255-1266 (2004).

8 Wang, X. *et al.* Influences from solvents on charge storage in titanium carbide MXenes. *Nat. Energy* **4**, 241-248 (2019).

9 Muckley, E. S. *et al.* Multimodality of Structural, Electrical, and Gravimetric Responses of Intercalated MXenes to Water. *ACS Nano* **11**, 11118-11126 (2017).

10 Xu, K. *et al.* Computational Insights into Charge Storage Mechanisms of Supercapacitors. *Energy Environ. Mater.* **3**, 235-246 (2020).

11 Xu, K. *et al.* Effects of functional groups and anion size on the charging mechanisms in layered electrode materials. *Energy Storage Mater.* **33**, 460-469 (2020).

12 Li, T. *et al.* Fluorine-Free Synthesis of High-Purity Ti_3_C_2_T*_x_* (T=OH, O) via Alkali Treatment. *Angew. Chem. Int. Ed.* **57**, 6115-6119 (2018).

13 Halim, J. *et al.* X-ray photoelectron spectroscopy of select multi-layered transition metal carbides (MXenes). *Appl. Surf. Sci.* **362**, 406-417 (2016).

14 Biesinger, M. C. *et al.* Resolving surface chemical states in XPS analysis of first row transition metals, oxides and hydroxides: Cr, Mn, Fe, Co and Ni. *Appl. Surf. Sci.* **257**, 2717-2730 (2011).

15 Yeung, K. L. *et al.* The influence of surface properties on the photocatalytic activity of nanostructured TiO_2_. *J. Catal.* **219**, 107-116 (2003).

16 Shahzad, A. *et al.* Heterostructural TiO_2_/Ti_3_C_2_T_x_ (MXene) for photocatalytic degradation of antiepileptic drug carbamazepine. *Chem. Eng. J.* **349**, 748-755 (2018).

17 Debeila, M. A., Coville, N. J., Scurrell, M. S. & Hearne, G. R. The effect of calcination temperature on the adsorption of nitric oxide on Au-TiO_2_: Drifts studies. *Appl. Catal. A: Gen.* **291**, 98-115 (2005).

18 Xu, S. *et al.* Binder-free Ti_3_C_2_T_x_ MXene electrode film for supercapacitor produced by electrophoretic deposition method. *Chem. Eng. J.* **317**, 1026-1036 (2017).

19 Lu, C. *et al.* Nitrogen‐Doped Ti_3_C_2_ MXene: Mechanism Investigation and Electrochemical Analysis. *Adv. Funct. Mater.* **30**, 2000852 (2020).

20 Chen, X. *et al.* The effect of postnitridation annealing on the surface property and photocatalytic performance of N-doped TiO2 under visible light irradiation. *J. Catal.* **255**, 59-67 (2008).

21 Lukatskaya, M. R. *et al.* Ultra-high-rate pseudocapacitive energy storage in two-dimensional transition metal carbides. *Nat. Energy* **2**, 17105 (2017).

22 Ghidiu, M., Lukatskaya, M. R., Zhao, M. Q., Gogotsi, Y. & Barsoum, M. W. Conductive two-dimensional titanium carbide 'clay' with high volumetric capacitance. *Nature* **516**, 78-81 (2014).

23 Li, K. *et al.* All-pseudocapacitive asymmetric MXene-carbon-conducting polymer supercapacitors. *Nano Energy* **75**, 104971 (2020).

24 Acerce, M., Voiry, D. & Chhowalla, M. Metallic 1T phase MoS2 nanosheets as supercapacitor electrode materials. *Nat. Nanotechnol.* **10**, 313-318 (2015).

25 Lin, T. *et al.* Nitrogen-doped mesoporous carbon of extraordinary capacitance for electrochemical energy storage. *Science* **350**, 1508 (2015).

26 Feng, D. *et al.* Robust and conductive two-dimensional metal−organic frameworks with exceptionally high volumetric and areal capacitance. *Nat. Energy* **3**, 30-36 (2018).

27 Tang, J. *et al.* Optimizing Ion Pathway in Titanium Carbide MXene for Practical High‐Rate Supercapacitor. *Adv. Energy Mater.*, 2003025 (2020).

28 Li, Z. *et al.* Tuning the interlayer spacing of graphene laminate films for efficient pore utilization towards compact capacitive energy storage. *Nat. Energy* **5**, 160-168 (2020).

29 Yang, C. *et al.* Flexible Nitrogen-Doped 2D Titanium Carbides (MXene) Films Constructed by an Ex Situ Solvothermal Method with Extraordinary Volumetric Capacitance. *Adv. Energy Mater.* **8**, 1802087 (2018).

30 Yang, C. *et al.* Methanol and Diethanolamine Assisted Synthesis of Flexible Nitrogen-Doped Ti3C2 (MXene) Film for Ultrahigh Volumetric Performance Supercapacitor Electrodes. *ACS Applied Energy Materials* **3**, 586-596 (2020).

31 Yang, C. *et al.* Improved capacitance of nitrogen-doped delaminated two-dimensional titanium carbide by urea-assisted synthesis. *Electrochim. Acta* **225**, 416-424 (2017).

32 Yang, C. *et al.* Achieving of Flexible, Free-Standing, Ultracompact Delaminated Titanium Carbide Films for High Volumetric Performance and Heat-Resistant Symmetric Supercapacitors. *Adv. Funct. Mater.* **28**, 1705487 (2018).

33 Al-Temimy, A. *et al.* Impact of Cation Intercalation on the Electronic Structure of Ti_3_C_2_T*_x_* MXenes in Sulfuric Acid. *ACS Appl. Mater. Interfaces* **12**, 15087-15094 (2020).

34. C. L. Jia, M. Lentzen, K. Urban, Atomic-Resolution Imaging of Oxygen in Perovskite Ceramics. *Science* **299**, 870 (2003).

35. G. Algara-Siller, O. Lehtinen, F. C. Wang, R. R. Nair, U. Kaiser, H. A. Wu, A. K. Geim, I. V. Grigorieva, Square ice in graphene nanocapillaries. *Nature* **519**, 443-445 (2015).

36. W. Zhou, K. Yin, C. Wang, Y. Zhang, T. Xu, A. Borisevich, L. Sun, J. C. Idrobo, M. F. Chisholm, S. T. Pantelides, R. F. Klie, A. R. Lupini, The observation of square ice in graphene questioned. *Nature* **528**, E1-E2 (2015).

37. R. Ma, D. Cao, C. Zhu, Y. Tian, J. Peng, J. Guo, J. Chen, X.-Z. Li, J. S. Francisco, X. C. Zeng, L.-M. Xu, E.-G. Wang, Y. Jiang, Atomic imaging of the edge structure and growth of a two-dimensional hexagonal ice. *Nature* **577**, 60-63 (2020).

38. J. Peng, D. Cao, Z. He, J. Guo, P. Hapala, R. Ma, B. Cheng, J. Chen, W. J. Xie, X.-Z. Li, P. Jelínek, L.-M. Xu, Y. Q. Gao, E.-G. Wang, Y. Jiang, The effect of hydration number on the interfacial transport of sodium ions. *Nature* **557**, 701-705 (2018).

39. J. Guo, X. Meng, J. Chen, J. Peng, J. Sheng, X.-Z. Li, L. Xu, J.-R. Shi, E. Wang, Y. Jiang, Real-space imaging of interfacial water with submolecular resolution. *Nature Mater.* **13**, 184-189 (2014).

40 Chong, S.-H. & Ham, S. Anomalous Dynamics of Water Confined in Protein–Protein and Protein–DNA Interfaces. *J. Phys. Chem. Lett.* **7**, 3967-3972 (2016).

41 Fumagalli, L. *et al.* Anomalously low dielectric constant of confined water. *Science* **360**, 1339 (2018).

42 Gopinadhan, K. *et al.* Complete steric exclusion of ions and proton transport through confined monolayer water. *Science* **363**, 145 (2019).

43 Raviv, U., Laurat, P. & Klein, J. Fluidity of water confined to subnanometre films. *Nature* **413**, 51-54 (2001).

44 Agrawal, K. V., Shimizu, S., Drahushuk, L. W., Kilcoyne, D. & Strano, M. S. Observation of extreme phase transition temperatures of water confined inside isolated carbon nanotubes. *Nat. Nanotechnol.* **12**, 267-273 (2017).
